# Transcriptional mutagenesis of α-synuclein caused by DNA oxidation in Parkinson’s disease pathogenesis

**DOI:** 10.1007/s00401-023-02632-7

**Published:** 2023-09-23

**Authors:** Sambuddha Basu, Minkyung Song, Levi Adams, Inhye Jeong, Goun Je, Subhrangshu Guhathakurta, Jennifer Jiang, Nikpreet Boparai, Wei Dai, Fernando Cardozo-Pelaez, Suren A. Tatulian, Kyu Young Han, Jordan Elliott, Jean Baum, Pamela J. McLean, Dennis W. Dickson, Yoon-Seong Kim

**Affiliations:** 1 Department of Neurology, Robert Wood Johnson Medical School, Institute for Neurological Therapeutics at Rutgers, Rutgers Biomedical and Health Sciences, 683 Hoes Lane West, Piscataway, NJ 08854 USA; 2 Burnett School of Biomedical Sciences, UCF College of Medicine, University of Central Florida, Orlando, FL 32827 USA; 3 Department of Cell Biology and Neuroscience, Institute for Quantitative Biomedicine, Rutgers University, Piscataway, NJ 08854 USA; 4 Center for Environmental Health Sciences, University of Montana, Missoula, MT 59812 USA; 5 Department of Physics, University of Central Florida, Orlando, FL 32816 USA; 6 CREOL, The College of Optics and Photonics, University of Central Florida, Orlando, FL USA; 7 Department of Chemistry and Chemical Biology, Rutgers University, Piscataway, NJ 08854 USA; 8 Department of Neuroscience, Mayo Clinic, 4500 San Pablo Rd, Jacksonville, FL 32224 USA; 9 Center for Structural and Functional Neurosciences, University of Montana, Missoula, MT 59812 USA

**Keywords:** 8-oxodG, Transcriptional mutagenesis, Alpha-synuclein aggregation, Lewy bodies, Parkinson’s disease

## Abstract

Oxidative stress plays an essential role in the development of Parkinson’s disease (PD). 8-oxo-7,8-dihydroguanine (8-oxodG, oxidized guanine) is the most abundant oxidative stress-mediated DNA lesion. However, its contributing role in underlying PD pathogenesis remains unknown. In this study, we hypothesized that 8-oxodG can generate novel α-synuclein (α-SYN) mutants with altered pathologic aggregation through a phenomenon called transcriptional mutagenesis (TM). We observed a significantly higher accumulation of 8-oxodG in the midbrain genomic DNA from PD patients compared to age-matched controls, both globally and region specifically to α-SYN. In-silico analysis predicted that forty-three amino acid positions can contribute to TM-derived α-SYN mutation. Here, we report a significantly higher load of TM-derived α-SYN mutants from the midbrain of PD patients compared to controls using a sensitive PCR-based technique. We found a novel Serine42Tyrosine (S42Y) α-SYN as the most frequently detected TM mutant, which incidentally had the highest predicted aggregation score amongst all TM variants. Immunohistochemistry of midbrain sections from PD patients using a newly characterized antibody for S42Y identified S42Y-laden Lewy bodies (LB). We further demonstrated that the S42Y TM variant significantly accelerates WT α-SYN aggregation by cell and recombinant protein-based assays. Cryo-electron tomography revealed that S42Y exhibits considerable conformational heterogeneity compared to WT fibrils. Moreover, S42Y exhibited higher neurotoxicity compared to WT α-SYN as shown in mouse primary cortical cultures and AAV-mediated overexpression in the *substantia nigra* of C57BL/6 J mice. To our knowledge, this is the first report describing the possible contribution of TM-generated mutations of α-SYN to LB formation and PD pathogenesis.

**Supplementary Information:**

The online version contains supplementary material available at 10.1007/s00401-023-02632-7.

## Introduction

Parkinson’s disease (PD) is the second most prevalent neurodegenerative disorder after Alzheimer’s disease (AD). PD is an age-related progressive disease characterized by loss of dopamine (DA) producing neurons in the substantia nigra pars compacta region (SNpc) of the mid-brain [3, 45]. In PD, reactive oxygen species (ROS) generated by mitochondrial dysfunction and dopamine metabolism render dopaminergic neurons susceptible to degeneration [45]. Pathologically, PD is characterized by cytoplasmic, eosinophilic inclusions called Lewy bodies (LBs) and Lewy neurites (LNs), which are composed of more than 90 proteins [20, 69, 76]. Alpha-synuclein (α-SYN) encoded by the gene *SNCA* is the principal protein of LBs attributed to its inherent aggregation potential to form β-sheet rich structures [69]. α-SYN plays a critical role in the pathogenesis of both familial and sporadic forms of PD and has thus drawn great attention in PD research to elucidate the molecular underpinnings of PD and therapeutic development [29, 40].

Earlier reports have strongly implicated increased oxidative stress as a key factor contributing to PD pathogenesis [72, 82]. Oxidative stress is accompanied by damage to cellular macromolecules like nucleic acid, protein, and lipid, whose function is indispensable for cell survival. The most common oxidative DNA lesion is 8-oxo-7,8-dihydroguanine (8-oxodG), which is attributed to the high oxidation susceptibility of guanine. It often underlies multiple disease conditions including neurodegeneration and cancer [1, 53, 56, 67]. 8-oxodG has strong mutagenic potential as this non-bulky DNA lesion could escape from specific repair machinery [6, 38, 65]. When DNA/RNA polymerase encounters 8-oxodG lesions in the DNA template strand, it can misincorporate adenine instead of cytosine through the Hoogsteen base pairing, causing G: C→T:A transversion mutation [16]. During transcription catalyzed by RNA polymerase II, 8-oxodG-induced misincorporation of adenine leads to transcriptional mutagenesis (TM) in a nascent mRNA, which eventually leads to the generation of mutated protein species. In post-mitotic cells like neurons, accumulation of TM-derived mutant species might contribute to neuronal degeneration [4–6]. Increased accumulation of 8-oxodG in both PD and AD patient brains compared to age-matched control subjects has been reported [1, 46, 54]. In this study, we tested whether 8-oxodG-mediated TM event generates novel pathological mutants of α-SYN with strong aggregation potential that drive template-directed misfolding of WT α-SYN, thus contributing to the α-SYN fibrillization process and pathogenesis of PD.

## Materials and methods

### Human post-mortem brain samples

Frozen human post-mortem midbrain tissues of controls and Parkinson’s disease patients were acquired from the NIH Neurobiobank and the Parkinson’s UK Brain Bank. The age ranges were from 54 to 89 years (average, 75.6 years old) for control subjects and 74 to 87 (average, 79.8 years old) for PD. The post-mortem interval (PMI) varied from 10.0 to 30.3 h (average, 19.6 h) for controls and from 3.0 to 26.0 h (average, 13.8 h) for PD cases. Formalin-Fixed Paraffin-Embedded (FFPE) serial sections of the midbrain of Parkinson’s disease (PD) were also procured from the NIH Neurobiobank. (Supplementary Table 1).

### Animals

An equal number of female and male C57BL/6 J mice (7 weeks, 18–20 g) were purchased from Jackson Laboratory (Bar Harbor, ME, USA). All animals were allowed to adjust to new environmental conditions for a week and were maintained on 12-h light/12-h dark at room temperature 20–23 °C with 40–60% humidity. Water and food were freely available. All animal experiments were performed in accordance with the ARRIVE guidelines and the relevant regulations and approved by the Institutional Animal Care and Use Committee of Rutgers University.

### Generation of α-SYN knockout HEK293T cell line using CRISPR-Cas9

α-SYN knockout HEK293T cells were generated by transfecting a cocktail of *SNCA-*specific sgRNA using Horizon’s free CRISPR guide program (Horizon discovery Ltd., UK). The sequence of sgRNA’s targeted towards the *SNCA* gene were 5′-AACAGGGTGTGGCAGAAGCA-3′, 5′-AGGAGGGAGTTGTGGCTGCT-3′, 5′-TTGAAAGTCCTTTCATGAAT-3′. The backbone vector containing the sgRNA had a fluorescent dasher-GFP, which was used for fluorescence-activated cell sorting.

(BD biosciences/Aria). The sorted GFP-containing cells were genotyped using the primers Forward-ACACCCCGAGTGAGCAGTTA and Reverse-CTGGAAAAGCAAACAGTCGC. Homozygous knockout of α-SYN protein was confirmed by immunoblotting with a specific antibody, mouse anti-α-SYN (1:500) (BD transduction #610,786).

### Cell culture and treatment paradigm

α-SYN knockout HEK293T cells were maintained in DMEM (Dulbecco′s Modified Eagle Medium) supplemented with heat-inactivated 10% fetal bovine serum and 1% Penicillin/Streptomycin (Gibco, 10,000 U/mL). Cells were maintained at 37 °C in humidified incubators with 5% CO_2_ and passaged following trypsinization with 0.25% Trypsin/0.53 mM EDTA (ThermoFisher Scientific). Transfections for all experiments were performed using X-fect polymer (CloneTech # 631,317) according to the manufacturer’s instructions.

### Analysis of 8-oxodG levels in human post-mortem brain samples (global and region-specific for α-SYN)

The 8-oxodG levels analysis from the frozen human post-mortem human brain samples were performed following the previous studies [8, 9]. Briefly, approximately 150 mg of frozen post-mortem SN sample was used to extract sufficient DNA for the assay. The purified DNA was digested with nuclease P1 followed by alkaline phosphatase treatment to yield deoxynucleoside for HPLC analysis. The amounts of 8-oxodG and 2-deoxyguanosine (2-dG) were calculated by comparing the peak area of 8-oxodG and 2-dG obtained from the enzymatic hydrolysate of the DNA sample to a calibration curve for both compounds. Levels of 8-oxodG in the samples were expressed relative to 2-dG (fmol 8-oxodG/nmol of 2-dG).

To measure the levels of 8-oxodG specific to the *SNCA* coding region, we developed a method called OxodIP (8-oxodG-DNA immunoprecipitation). First, we isolated genomic DNA from frozen post-mortem SN samples. The extracted DNA was then randomly digested with Tsp5091 (Fermentas #ER1351) and precipitated using an 8-oxodG-specific monoclonal antibody (Abcam N45.1). The immunoprecipitated DNA was amplified by PCR using primer sets for the coding region of *SNCA* (exon 5) to get an estimation of the 8-oxodG accumulation. Immunoprecipitated DNA was quantified using densitometric analysis against the input (digested DNA that was not immunoprecipitated).

### Analysis of OGG1 mRNA levels and activity in human post-mortem brain samples

Levels of OGG1 mRNA were analyzed using semi-quantitative reverse transcriptase PCR (RT-PCR). Briefly**,** approximately 50 mg of the frozen post-mortem brain containing the SNpc region was used for RNA extraction using Trizol reagent according to manufacturer protocols (Life Technologies Inc #cat no. 15596-026). Complementary DNA (cDNA) was generated by conversion of 1 µg total RNA using amfiRivert cDNA synthesis platinum master mix according to the manufacturer′s protocol (GenDEPOT #R5600-50). The synthesized cDNA was diluted 1:1 with nuclease-free water followed by PCR amplification with primers for human OGG1 (Forward-TGGAGTGGTGTACTAGCGGA, Reverse-GGATGAGCCGAGGTCCAAAA) and OGG1 expression was normalized by an endogenous control gene, β-actin (Forward-GGAGTCCTGTGGCATCCACG, Reverse- CTAGAAGCATTTGCGGTGGA).

OGG1 activity from the SN region of human post-mortem brain samples was performed in accordance with the published protocol [8]. Briefly, tissue was dissected on ice and placed under liquid nitrogen, and DNA glycosylases were extracted from tissue following homogenization with buffer containing 20 mM of Tris (pH 8.0), 1 mM of EDTA, 1 mM of dithiothreitol, 0.5 mM of spermine, 0.5 mM of spermidine, 50% glycerol, and protease inhibitors. Homogenates were rocked for 30 min after the addition of a 1/10 vol/vol of 2.5 M of KCl and spun at 14,000 rpm for 30 min. The supernatant was aliquoted and stored at  – 70 °C until the time of assay. Protein levels were determined using the BCA method. 20 pmol of a synthetic probe containing 8-oxodG (Trevigen, Gaithersburg, MD, USA) was labeled with P^32^ at the 5′ end using polynucleotide T4 kinase (Boehringer Mannheim, Germany). For the nicking reaction, protein extract (30 μg) was mixed with 20 μl of a reaction mixture containing 0.5 M of N-[2-hydroxyethyl] piperazine-N′-[2-ethanesulfonic acid], 0.1 M of EDTA, 5 mM of dithiothreitol, 400 mM of KCl, purified BSA, and labeled probe (approximately 2000 cpm). The reaction was carried out at 37 °C for 2 h and stopped by placing the solution in ice. An aliquot of this reaction mixture was added to a loading buffer containing 90% formamide, 10 mM of NaOH, and blue-orange dye. After 5 min of heating at 95 °C, samples were chilled and loaded into a polyacrylamide gel (20%) with 7 M of urea and 1 × TBE and run at 400 mV for 2 h. Gels were quantified using a Biorad (Hercules, CA, USA) 363 phospho-imager system and analysis software. Activity to repair 8-oxodG was determined and expressed as a percentage of substrate cleaved.

### RNaseH2 PCR for detection of each TM variant

Primers for the RNaseH2 PCR (rhPrimers) were designed following the manufacturer’s instruction (Integrated DNA Technologies #11-02-12-01) to detect the TM-generated α-SYN variants. Each mutant was detected using 2nd generation rhPrimers which were designed and validated to specifically detect the respective mutant mRNA. For each of the mutant primers designed, we designed a corresponding WT primer (with WT sequence at the position of the mutation). PCR amplification of each mutant mRNA species (L38I, S42Y, H50N, A53E, T72K, T75K, and S129Y) was done from cDNA synthesized (as described above) using 2 μg of total RNA extracted from human post-mortem control and PD SN samples. For each PCR reaction, 120 milli-units of the RNaseH2 enzyme was used along with 10 pM of the forward and the reverse primers. The PCR condition was optimized at 57 °C annealing temperature and 38–40 cycles. For each PCR reaction from plasmids used for validation of the primers, 0.5 milli-units of the RNaseH2 enzyme was used along with 1 pM of the forward and the reverse primers. For the validation of RNaseH2 PCR, 1 μg of full-length WT and S42Y α-SYN (as described in the plasmid section) was transfected into α-SYN knockout HEK293T cells. At 48 h post-transfection, cells were harvested and followed by extraction of RNA and cDNA (Complementary DNA) synthesis. rhPrimers used for RNaseH2 PCR are listed in a Supplementary Table 2.

### α-SYN coding region-specific amplicon-sequencing

Total RNA was extracted from 5 controls and 8 PD midbrain samples using Trizol (Invitrogen, MA, USA), and genomic DNA was extracted from 4 controls and 3 PD samples using the DNA miniprep kit (Qiagen, Venlo, Netherlands). The mRNA coding regions, spanning 423 bp, were fully covered. The genomic location was designed to amplify a 219-nucleotide-long DNA template containing the S42Y and A53E regions. AmpliconEZ from GENEWIZ was used for NGS sequencing using the following primers: cDNA Forward-ATGGATGTATTCATGAAAGGACTTTC, cDNA Reverse-GGCTTCAGGTTCGTAGTCTT, gDNA Forward-ACTAGCTAATCAGCAATTTAAGGCT, and gDNA Reverse-TGTTCTTAGAATGCTCAGTGATTG. PCR amplification was performed using high-fidelity DNA polymerase (Platinum Superfii PCR master mix; Invitrogen, MA, USA). The presence and size of each amplicon were confirmed by gel electrophoresis using a 2% agarose gel. Amplified fragments were subsequently purified using the PCR Purification Kit (Thermo Scientific, MA, USA). The double-stranded DNA (dsDNA) was quantified using Qubit, normalized to a concentration of 20 ng/µL, with a total amount of 500 ng. Following DNA library preparation, sequencing reactions and data processing by GENEWIZ, over 300,000 reads were aligned to the reference sequence for low-frequency variant detection. The variant allele frequency (VAF) cutoff was set at 0.1%.

### Sequencing of the amplified product from the cDNA and the genomic DNA

The detected TM α-SYN variants by RnaseH2 PCR from the human post-mortem PD and control brain samples were gel extracted and sequenced after cloning it into pGEMT-Easy vector system I (Promega # A1360). The same region of the genomic DNA was amplified (Forward-ATTCGACGACAGTGTGGTGTAAAG and Reverse-TCCACAGGCATATCTTCCAGAAT) and cloned into pGEMT-Easy vector system I. 5–10 colonies from each of the cloned products were sequence verified to confirm the presence of the mutation in the cDNA but not in the genomic DNA.

### Generation of plasmids containing WT, A53T, and S42Y α-SYN

pAAV-3xFlag-hα-SYN-IRES-hrGFP constructs were established by the two-step cloning. First, human α-SYN coding region was amplified and inserted into the p3XFLAG-myc-CMV-23 (Sigma, #E6026) [11, 15]. Next, the region containing 3xFalg-WT hα-SYN was subcloned into the pAAV-IRES-hrGFP plasmid (Agilent technologies #240,075). S42Y and A53T mutation was introduced using a Quick-change site-directed mutagenesis kit (Agilent technologies #210,515) using mutation-specific primers. The primers used for generating the S42Y and A53T α-SYN were (S42Y: Forward-TATGTAGGCTACAAAACCAAGGA Reverse-TCCTTGGTTTTGTAGCCTACATA) and (A53T: Forward-GTGCATGGTGTGACAACAGTGGCT and Reverse- AGCCACTGTTGTCACACCATGCAC). The sequence of each construct was confirmed by sequencing.

Recombinant α-SYN protein was generated using bacterial expression plasmids. pT7-7 vector containing WT hα-SYN was received as a gift from Hilal Lashuel (Addgene plasmid # 36,046) [60]. pT7-7 S42Y hα-SYN was established by a site-directed mutagenesis reaction using the primers described above and a quick-change site-directed mutagenesis kit (Agilent technologies #210,515).

The constructs for the split luciferase-based complementation assay system containing full-length WT α-SYN (α-SYN-hGLuc1 (S1) and α-SYN-hGLuc2 (S2)) were generated, as described previously [59], by subcloning α-SYN into the Not1/ClaI sites of humanized Gaussia luciferase constructs, and kindly provided by Dr. Nicklaus McFarland of the University of Florida.

### Single-molecular pull-down (SiMPull) assay to validate anti-S42Y α-SYN antibody

Rabbit anti-S42Y α-SYN antibody was generated by Abcam. Briefly, both WT (GVLYVGS*KTKE) and S42Y (GVLYVGY*KTKE) peptides of 11 amino acids spanning the region containing S42 were synthesized. Arginine and cysteine were added at the N-terminal for solubility and conjugation, respectively. Four rabbits were immunized 5 times with 0.2–0.4 mg of mutant peptide every two weeks. Antiserums were collected 1 week after 3rd and the last immunization for the measurement of specificity and titers. Antiserum exhibiting high specificity against mutant α-SYN was subjected to purification using an S42Y peptide affinity and WT peptide depletion column. To validate the specificity of anti-S42Y α-SYN antibody, a SiMPull assay was performed based on our previous study [33]. Briefly, passivated quartz slides and coverslips were assembled into flow chambers. After washing with PBS (pH 7.4), 40 µL of 0.2 mg/mL NeutrAvidin (Thermo Fisher Scientific) in PBS was introduced for 5 min, then washed with 100 µL of wash buffer (0.1 mg/mL BSA in PBS). Similarly, after introducing 5 µg/ml biotinylated anti-mouse IgG antibody (Abcam, ab97033) and 2.5 µg/ml mouse monoclonal α-SYN antibody (BD Biosciences, 610,786), 40 µL of appropriately diluted recombinant S42Y or mixtures of S42Y and WT α-SYN incubated overnight with 1.3 µg/ml rabbit anti-S42Y α-SYN antibody (Abcam) were loaded and incubated for 30 min on antibody-tethered coverslips. Unbound components were washed out with 100 µL of wash buffer and then 0.7 µg/ml Alexa 647-labeled anti-rabbit full IgG (Invitrogen, A31573) was introduced for 5 min. Images were captured by a custom-made objective-type total internal reflection fluorescence (TIRF) microscope [70] with an imaging buffer containing 0.8% (w/v) dextrose (Sigma), 1 mg/mL glucose oxidase (Sigma), 0.04 mg/mL catalase (EMD Millipore) and 2 mg/mL Trolox (Santa Cruz) to minimize photobleaching of Alexa 647. At least more than 20 images were acquired at different locations and each imaged area was ~ 4,700 µm^2^. The number and fluorescence intensity of pull-down molecules were analyzed with MATLAB scripts similar to previous studies [31, 70].

### Immunoblotting for analysis of soluble and aggregated fractions of α-SYN

For western blotting, total protein was extracted using RIPA buffer (PBS, 1% NP-40, 0.5% Sodium deoxycholate, 0.1% PMSF, 100 ng/ml protease inhibitor, dH2O), and 40 μg of total protein lysate was run on 10% denaturing SDS gel and transferred to PVDF membrane (Millipore cat no. IPFL00010). Following the transfer process, the membrane was blocked with 5% non-fat dry milk in TBS-T (tris buffer saline with 0.1% tween-20) before incubation with primary antibodies.

The aggregation potential for each mutant was determined following the treatment conditions published with slight modifications [37, 58, 77]. For the cell-based biochemical analysis of aggregation, α-SYN knock out HEK293T cells were transfected with a total 5 μg of empty backbone vector, WT, S42Y, or WT/S42Y α-SYN (in desired ratios) in a 6-well plate format. Following transfection, cells were treated with 300 μM of FeCl_2_ (Sigma Aldrich # 372,870**)** for 96 h. Fresh media supplemented with 300 μM of FeCl_2_ was replenished after 48 h. On the last day before further processing, cells were treated with 5 μM of MG132 for 6 h.

For detergent solubility-based analysis of the aggregated fraction of α-SYN, we followed a published protocol with slight modifications [77]. Briefly, the total cell pellet was incubated with triton X-100 (final concentration 1% in TBS containing protease inhibitor cocktail) for one-hour rotation at 4 °C followed by centrifugation (16,000 × g, 30 min, 4 °C). The supernatant was designated as a triton X-100 soluble fraction. Triton X-100 insoluble pellet was washed three times with ice-cold TBS by rotating at 4 °C followed by centrifugation at 16,000 × g for 5 min to remove residual soluble fraction. Then, the pellet was dissolved in 2% SDS in TBS and sonicated for 30 s (2 s each with 2 s intervals till the pellet dissolved). This fraction was designated as a triton X-100 insoluble fraction. Protein concentration was determined using a Lowry protein assay. For the soluble fraction, 15 μg of denatured protein was loaded onto 10% SDS-gels for western blot analysis, and for the aggregated fraction, 50 μg of protein was run. Gel running was performed with SDS-containing running and sample loading buffer according to standard procedures.

The higher molecular weight bands of α-SYN were evaluated using a non-denaturing condition, where the protein was not boiled at 95 °C for 5 min. For analysis of the recombinant protein, 2–5 μg each of the recombinant protein was run on 10% SDS-containing gel following standard procedures. The antibodies used for the western blot analysis were mouse anti- α-SYN antibody (BD transduction lab #610,786), rabbit polyclonal anti-S42Y α-SYN antibody (developed from Abcam), rabbit anti-pS129 α-SYN antibody (Santa Cruz # sc135638), and mouse anti-β-actin (Sigma # A2228).

### Bioluminescence split complementation assay

Bioluminescence split complementation assay with the different mutants was performed using α-SYN-hGLuc1 (S1) and α-SYN-hGLuc2 (S2) constructs based on the principle reported in the publication [59, 64]. Briefly, equal numbers of α-SYN knock out HEK293T cells were seeded in 24-well plate in triplicates. For each well, 500 ng of the individual α-SYN containing split luciferase constructs (S1 + S2) were transfected along with 10 ng of the WT or mutant α-SYN constructs in a 1:100 ratio. At each time point, the media was assayed for luciferase activity using BioLux® *Gaussia* Luciferase Assay Kit (NEB# E3300S) following the manufacturer’s protocol.

### Immunocytochemistry and proteinase-K-resistant staining

For immunostaining and proteinase-K resistant staining of α-SYN aggregates, cells after undergoing the aggregation protocol (described above) were fixed in 4% paraformaldehyde (PFA) for 20 min and permeabilized with 0.1% triton X-100 in PBS for 10 min. Blocking was performed by incubation with 10% donkey serum in PBS containing 0.1% tween-20 for 1 h at room temperature. The cells were then incubated with mouse anti-α-SYN (1:500) (BD transduction #610,786). After washing, the cells were incubated with the appropriate anti-mouse antibody conjugated to Alexa Fluor 546 (1:1000). For nuclear visualization, cells were incubated with 2 μM Hoechst for 15 min.

The proteinase-K (PK) resistant staining for α-SYN aggregation was followed according to our previous study [15]. Briefly, 10 ug/mL of PK was treated in the cells for 30 min at 37 °C. PK was then inactivated with 3 M guanidine-thiocyanate in 10 mM Tris–HCl solution for 10 min. Between each step, the cells were gently washed with PBS three times. α-SYN was visualized as described above. For quantification of the PK-resistant α-SYN aggregates, punctate aggregated structures were counted from 5 to 8 fields under each experimental condition [61].

### ThT fluorescence assays

WT α-SYN was expressed and purified as described previously [81]. The S42Y mutant was produced as described above, with subsequent analysis carried out via mass spectrometry and SDS-PAGE. Purified α-SYN (WT or S42Y) samples were initially passed through 50-kDa filters to remove aggregates and then concentrated by using a 3-kDa filter (Millipore Sigma, St. Louis, MO). Concentrated samples were diluted with PBS (10 mM, pH = 7.4), mixed with 20 μM ThT (Acros Organics, Pittsburgh, PA), and loaded into 96-well plates (Corning, Corning, NY). ThT fluorescence intensity (480 nm) was measured every 33 min by a POLAR Star Omega plate reader (BMG Labtech, Cary, NC). Monomer assays were conducted at 37 °C with the addition of a Teflon bead (3 mm; Saint-Gobain N.A., Malvern, PA) to each well, with shaking at 600RPM. Seeded assays were conducted by combining seeds made from sonicated fibrils at a concentration of 1% monomer equivalents with the addition of 35 µM monomer. Seeded assays were conducted in PBS (10 mM, pH = 7.4) at 37 °C in the absence of Teflon beads, under quiescent conditions (no shaking), with a brief mixing sequence after each reading cycle (100 RPM, linear). Each trace represents averages for at least 3 replicate wells.

### Mouse primary cortical neuron culture

Primary C57BL/6 neuronal cultures were established as previously described [68] with minor modifications. Briefly, brains were isolated from p16.5 embryonic pups and placed in 1 × HBSS supplemented with 1X Pen/Strep and 6 mg/mL D-glucose. After meninges were removed, the cortex was isolated, minced, and digested with 0.05% Trypsin–EDTA (Hyclone) containing DNAse I (200U/mL) at 37 °C for 10 min, and neutralized with 10% FBS (Gemini) in Neurobasal medium (Gibco). Cells were triturated with fire-polished Pasteur pipettes and resuspended in Neurobasal media containing 2% B27 (Gibco), 1X Pen/Strep, and 1X Glutamax (Gibco). Neurons were seeded at a concentration of 1 × 10^5^ cells/cm^2^ onto culture vessels coated with PEI and Laminin. After waiting 24 h for cells to attach, 1 uM arabinose-C was added to neurons for 48 h to restrict glial growth, followed by a full media change. Weekly thereafter, neurons received 50% media change for maintenance.

### α-SYN pre-formed fibril (PFF) treatment

To generate PFF, recombinant protein stocks (5 mg/ml) were placed on an orbital shaker for 7 days at 1000 rpm at 37 °C, then stored at  – 80 °C until ready to use. Immediately before treatment, PFF stocks were diluted to 500 ug/mL in DPBS and sonicated (30% Amplitude, 1 s alternating on/off pulse, 60 s total). Primary neurons were treated with sonicated fibrils at a concentration of 10 µg/mL. Immediately after PFF treatment, primary cultures were placed in an Incucyte (Sartorius, IC50728) and imaged at 20X every 8 h for the treatment period.

### Packaging and stereotaxic injection of AAV containing α-SYN in the SN

Viral particles (AAV2) were produced at the University of Iowa Viral Vector Core according to their standard operating procedures (https://medicine.uiowa.edu/vectorcore/). Mice were randomly divided into 2 groups (n = 5 per group): pAAV-WT hα-Syn group and pAAV-S42Y hα-Syn group. For injection of AAV constructs, the mice were deeply anesthetized with 3% isoflurane and placed in a stereotactic instrument (Stoelting, IL, USA). 2 uL of AAV particles (2 × 10^12^ viral genomes/mL) containing WT or S42Y hα-SYN were stereotaxically injected into the right SN with a coordinate of anteroposterior (AP) -3.0 mm, mediolateral (ML) -1.4 mm, and dorsoventral (DV)  – 4.4 mm from bregma. After 14 days, mice were sacrificed by cardiac perfusion of saline followed by fixation with 4% paraformaldehyde (0.1 M PB, pH 7.4).

### Immunostaining

For immunocytochemical staining for the primary cultures, cells were fixed with 4% PFA for 10 min followed by permeabilization with 0.2% TritonX-100 for 10 min. Primary antibodies (rabbit anti-GFAP, DAKO #Z0334, 1:500; rabbit anti-MAP2, Proteintech #17,490–1-AP, 1:500; rabbit anti-NeuN, Millipore #ABN78, 1:500; rabbit anti-p62, LSBio #LS-B4617, 1:200; rabbit anti-γH2A.X, Cell Signaling #9718S, 1:200; rabbit anti-p62, LSBio #LS-B4617, 1:200) were treated overnight at 4 °C, and Alexa Fluor 488 goat anti-rabbit secondary antibody (1:500) for 1 h at room temperature. Following immunostaining, nuclei were visualized by staining with 1 µM Hoescht for 10 min.

For mouse immunohistochemistry, fixed brains were saturated with 30% sucrose and sectioned in OCT media at 30 µm thickness. Endogenous peroxidase activity was quenched using 0.3% hydrogen peroxide followed by blocking in 1% BSA before incubation in primary antibody (mouse anti-Flag, Sigma #F3165, 1:1,000; rabbit anti-Iba-1, Abcam #ab178846, 1:500; rabbit anti-pS129, Abcam #ab51253, 1:200; mouse anti-TH, Santa Cruz #sc-25269, 1:500; rabbit anti-γH2A.X, Cell Signaling #9718S, 1:200; rabbit anti-S42Yα-SYN, Abcam, custom-made 1:200) overnight at 4 °C, and biotinylated goat anti-mouse or rabbit secondary antibodies for 1 h at room temperature. Visualization was performed using avidin–biotin complex (ABC) and DAB staining kits (Vector Biosciences) and sections were imaged Leica DMi8 at 20X.

Formalin-fixed paraffin-embedded (FFPE) human brain sections were deparaffinized and rehydrated before antigen retrieval (Sodium citrate buffer pH 6.0, 95–100 °C for 30 min). For 8-oxodG staining, slides were treated with RNase A (10 mg/mL, 37 °C for 30 min). Tissue sections were blocked in 1% BSA before incubation in primary antibody (anti-mouse 8-oxodG, JaICA #N45.1, 1:200; mouse anti-α-SYN, BD Transduction #610,786, 1:500; rabbit anti-S42Yα-SYN, Abcam, custom-made, 1:200; mouse anti-Ubiquitin, Chemicon #MAB1510, 1:250) overnight at 4 °C, and biotinylated goat anti-mouse secondary antibody (1:500) or alkaline phosphatase (AP) conjugated goat anti-rabbit secondary antibody (1:200) for 1 h at room temperature. Visualization was performed using ABC, DAB, and alkaline phosphatase staining kits (Vector Biosciences) according to the manufacturer’s instructions, and sections were imaged Leica DMi8 at 20X. Cresyl violent staining was performed after DAB staining according to the manufacturer’s instructions (FD Neurotechnologies Inc). Hematoxylin and Eosin staining was performed using an H&E Staining kit (Abcam) according to the manufacturer’s instructions.

### Cell-type-specific double immunofluorescence staining for 8-oxodG with signal amplification

Double immunofluorescence stainings were performed using monoclonal 8-oxodG (clone N45.1; Jaica, Japan) antibody together with polyclonal antibodies including TH, Olig2, GFAP, and Iba1. For human tissue, the deparaffinization and rehydration process was followed by a 15-min Heat-Induced Epitope Retrieval (HIER) step. Acid hydrolysis was performed by incubating samples in 2N HCl for 10 min at room temperature, followed by neutralization with a 50 mM Tris-base buffer. Endogenous peroxidase activity was quenched by 3% hydrogen peroxidase treatment for 10 min and non-specific binding was blocked using Trueblack (Biotium, CA, USA) for 1 min at room temperature (RT). A mixture of 8-oxodG mouse monoclonal antibody (1:200 dilution) and rabbit polyclonal antibody (1:500 dilution) was incubated on slides overnight at 4 °C. HRP mouse secondary antibody and fluorescent rabbit secondary antibody were mixed and applied for 1 h. Styramide labeling was performed using styramide working solution (AAT Bioquest, CA, USA) for 10 min at RT. Slides were counterstained with Hoechst and mounted. Images were acquired using a confocal microscope (Dragonfly, Belfast, Northern Ireland), and quantification was performed using Imaris software.

### Cryo-electron tomography for WT and S42Y fibrils

#### Preparation of EM Grids

WT and S42Y αSyn preformed fibril samples were mixed with 10 nm gold particles as fiducial markers to facilitate tilt series alignment. An aliquot of 3.5 μL of each sample was applied to glow-discharged Quantifoil holey grids (R2.0/1.0, Cu, 200 mesh; Quantifoil) prior to vitrification using a Leica EM GP plunger (Leica Microsystems, Buffalo Grove, IL, USA) in a humidity (95%) and temperature (20 °C) controlled chamber. Plunge-frozen grids were stored in a liquid nitrogen dewar flask until imaging.

#### Tomography data collection and data processing

Tilt series of the S42Y fibril sample were collected on a Talos Arctica cryo-electron microscope (Thermo Fisher Scientific, Waltham, MA, USA) operated at 200 kV, equipped with a post-column BioQuantum energy filter (the slit was set to 20 eV) and a K2 direct electron detector. Automated data collection was performed using SerialEM under the following conditions: 63,000 × microscope magnification, spot size 7, 100-μm condenser aperture, and defocus range of 6 ~ 3 µm. The image pixel size was 2.134 Å/pixel. Tilt series ranged from − 60° to 60° at 3° step increments. A total of 35 tilt series were collected in counting mode with a cumulative dose of 80–100 e-/Å2. For the WT preformed fibrils, we collected tilt series at 49,000 × microscope magnification, the spot size of 8, and defocus of  – 4 μm. All other imaging parameters were the same as described for the S42Y data collection. Tilt series alignment and tomogram reconstruction were performed using the latest EMAN2 tomography workflow [10]. Measurements of the fibril species were done using the EMAN2 measurement functionality. Subtomogram analysis and visualization of the tomogram were done using Chimera (University of California, San Francisco) [62].

### Statistical analysis

To get statistically meaningful data, all experiments were performed in technical triplicates. Statistical analysis was performed using Graph pad prism 9.3.1 software. Statistical significance of total TM load of α-SYN between control subjects and PD was assessed by a Pearson’s chi-square test of independence, examining the relationship between Parkinson’s disease and TM incidence. To test primary cortical neuronal death over 7 consecutive days after WT and S42Y α-SYN PFF treatments, two-way ANOVA was performed followed by Tukey’s post hoc tests for multiple comparisons. For comparing between 2 groups, data were analyzed using the Mann–Whitney test or the Student’s *t*-test. To compare among 3 groups, one-way ANOVA followed by Tukey’s post hoc test was performed. Data represent mean ± standard error of the mean (S.E.M). *p < 0.05; †*p* < 0.01; ‡*p* < 0.001; §*p*, 0.0001; n.s., non-significant. To determine the correlation between 8-oxodG level and OGG1 activity, two-tailed Pearson’s correlation was used followed by linear regression analysis. Values of *p* < 0.05 were considered significant.

## Results

### Increased 8-oxodG accumulation in the SNpc region of PD brain both in global and region-specific to *SNCA*

**Fig. 1:**
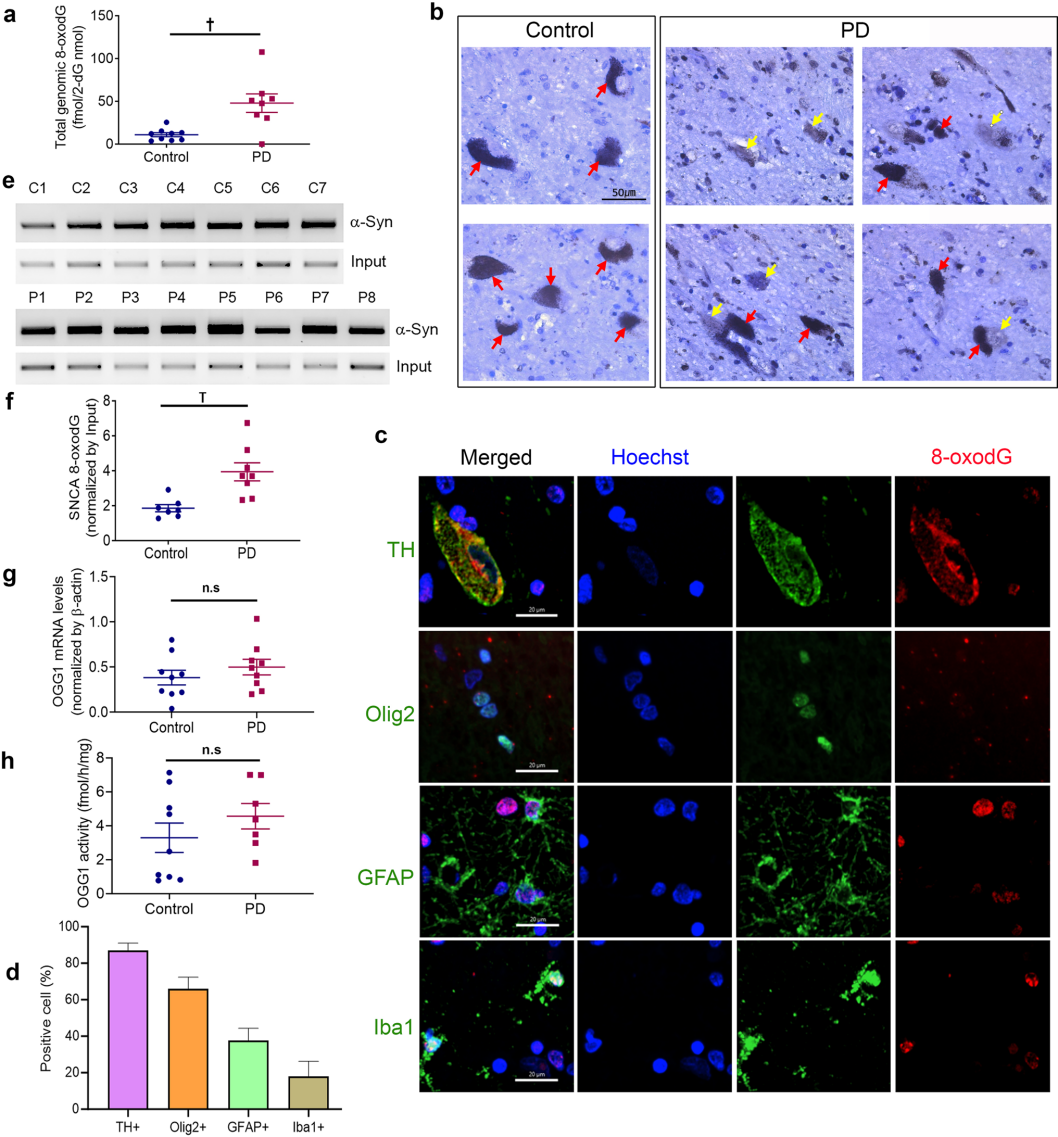
8-oxodG is significantly accumulated in the midbrain genomic DNA of Parkinson’s disease patients compared to age-matched controls.** a** Analysis of genomic DNA from the substantia nigra of PD postmortem brain samples (*n* = 8) showed a significant increase in 8-oxodG levels compared to age-matched control samples (*n* = 9). **b** Representative photomicrographs of 8-oxodG immunostaining in controls and PD midbrain samples, demonstrating increased 8-oxodG in PD compared to controls. To identify cell types, tissues were counter-stained with cresyl violet. 8-oxodG immunoreactivity is shown in dark grey after cresyl violet staining. Red arrows, dopaminergic neurons containing neuromelanin; yellow arrows, 8-oxodG stainings. **c** Representative confocal fluorescence images of double immunofluorescence stainings of 8-oxodG (red) with cell-type-specific markers (green) in PD midbrain samples. Dopaminergic neurons, oligodendrocytes, astrocytes, and microglia were visualized (green) with TH, Olig2, GFAP, and Iba1, respectively, together with 8-oxodG (red). Representative images of each immunofluorescence stainings from 4 PD midbrain samples containing the SN region. Scale bar = 20 µm. **d** Percentage of cells expressing nuclear 8-oxodG. n = 10 fields per staining. **e** Representative gel images of α-SYN Oxo-DIP analysis of PD and control midbrain samples. **f** Quantitative analysis of α-SYN Oxo-DIP, showing a significant increase in 8-oxodG levels on the exon 5 of *SNCA* of PD compared to controls. **g** Semi-quantitative RT-PCR showed no significant changes in OGG1 mRNA levels between the PD (*n* = 9) and control subjects (*n* = 9). **h** Similarly, analysis of OGG1 activity to cleave 8-oxodG containing oligonucleotide showed no significant difference in activity between the PD (*n* = 7) and control subjects (*n* = 9). Data represent mean ± SEM. † *p* < 0.01, *n.s.* non-significant

We investigated 8-oxodG accumulation in the genomic DNA from the SNpc region of PD and age-matched control *post-mortem* samples. As reported previously [1], we also observed a significant increase in the 8-oxodG levels in total genomic DNA purified from the midbrain of PD patients (*n* = 8) compared to the age-matched controls (*n* = 9) (*p* = 0.0079) (Fig. 1a). Immunostaining for 8-oxodG also confirmed this finding, showing apparent elevation in 8-oxodG staining in the SNpc of PD compared to young and age-matched controls (Fig. 1b, Supplementary Fig. 1a). Interestingly, 8-oxodG staining was generally increased not only in neuromelanin-containing dopaminergic neurons but also in other types of cells (Fig. 1c, d). 8-oxodG was detected in 87.1%, 65.9%, 37.8%, and 17.9% of dopaminergic neurons (TH +), oligodendrocytes (Olig2 +), astrocytes (GFAP +), and microglia (Iba1 +), respectively (Fig. 1c). We developed OxodIP (see Method) to further compare the 8-oxodG accumulation specifically in the *SNCA* coding region, and found significant increases in 8-oxodG in the *SNCA* of PD compared to controls (*p* = 0.0012) (Fig. 1e,f). This observation laid the foundation of the hypothesis to study the 8-oxodG-mediated generation of TM variants of α-SYN in PD.

To investigate the underlying mechanism implicated in this aberrant accumulation of 8-oxodG in PD, we investigated the expression and activity of 8-oxodG DNA glycosylase (OGG1), an enzyme responsible for specific 8-oxodG repair using the same cohorts used for the 8-oxodG analysis. There were no significant differences observed in OGG1 mRNA levels between the two groups, as indicated by semi-quantitative PCR (Fig. 1g). OGG1 activity for the ability to repair 8-oxodG lesion also showed no difference between controls and PD samples (Fig. 1h, Supplementary Fig. 1b). In a previous report, it was shown that only mitochondrial isoform of OGG1 expression was significantly changed in dopaminergic neurons in the SNpc between control and PD [23]. Interestingly, however, we observed a significant negative correlation between 8-oxodG levels and OGG1 activity only in the control group (*R*^2^ = 0.6356, *p* = 0.0102) but not in PD (*R*^2^ = 0.4797, *p* = 0.0864) (Supplementary Fig. 1c).

Taken together, these results show a significantly higher accumulation of 8-oxodG both global and specific to the *SNCA* coding region in PD, strongly supporting our hypothesis that 8-oxodG-mediated TM could contribute to PD pathogenesis. The result also implies that the increased 8-oxodG observed in the SNpc of PD was not due to the failure of OGG1 repair machinery.

### TM-generated α-SYN mRNA mutants in the human post-mortem PD midbrain

**Fig. 2:**
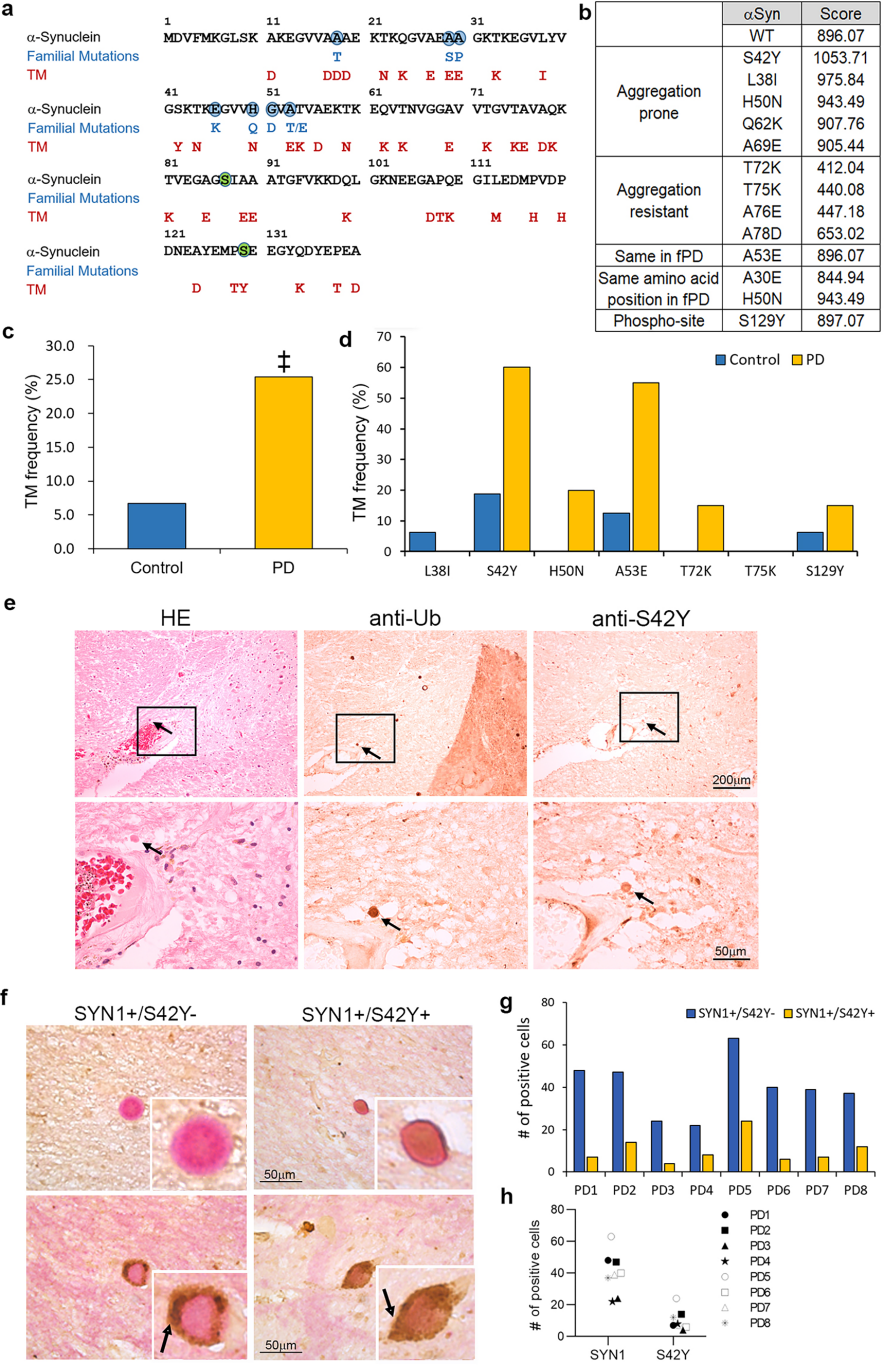
Detection of α-SYN TM variants both in mRNA and protein. α-SYN TM variants are increased in the midbrain of PD patients compared to controls and detected in some LBs.** a** Hypothetical α-SYN mutations caused by TM. All the possible mutant amino acid positions of α-SYN generated by 8-oxodG-driven TM are shown in red (Bottom). Mutations associated with familial PD are shown in blue (Middle). Blue circles indicate WT α-SYN amino acids mutated in familial PD. Green circles indicate serine residues that are subject to phosphorylation. **b** In silico analysis of the aggregation propensity of the α-SYN TM variants using TANGO. β-aggregation propensity score for the WT and some of the α-SYN TM variants are calculated. **c** Overall α-SYN TM mutant levels were significantly higher in PD than in control midbrain samples. χ^2^ = 14.186, ‡*p* < 0.001.** d** Frequency of each α-SYN TM variant varied between control and PD. (Control, *n* = 16; PD, *n* = 20) **e** Representative photomicrographs showing an LB immuno-positive for S42Y α-SYN. LBs were visualized using HE staining and anti-ubiquitin antibody together with anti-S42Y antibody in 6 µm-apart serial sections of PD midbrain. Black arrows, co-labeled LB. Lower panels, magnified boxed areas in the upper panels. **f** Double immunostaining of LBs for total α-SYN and S42Y variant. All LBs were visualized by α-SYN antibody (pink), and S42Y was detected using a specific antibody (brown). Black arrows, neuromelanin. The left column shows LBs negative for S42Y; the right column shows LBs co-stained with S42Y. **g,h** The total number of cells having LBs and S42Y-positive LBs for each PD individual (**g**) and together (**h**)

All possible TM-derived amino acid changes in α-SYN are predicted by replacing cytosine with adenine in the α-SYN mRNA strand, which could be misincorporated due to 8-oxodG in the DNA template strand (Fig. 2a) [4]. A total of 43 missense mutations can be introduced by the 8-oxodG-mediated TM event. 5 mutations at the same amino acid position of the known familial mutants (A18T, A29S, A30P, H50Q, and A53T/E) could occur with different amino acid residues (A18D, A29E, A30E, H50N, and A53E, respectively) as a result of TM. Intriguingly, A53E that was reported in familial PD could be generated by the TM event. We next used TANGO, a statistical mechanics algorithm, to predict the aggregation propensity of each α-SYN TM variant and compared it with the score of wild-type (WT) α-SYN (Fig. 2b, Supplementary Table 3) [21]. The TANGO algorithm indicated that some α-SYN TM variants have higher β-aggregation scores than WT α-SYN, which include L38I, S42Y, H50N, Q62K, and A69E. These five variants are located in either the N-terminal (1–61 amino acid) or the NAC (non-Aβ component of amyloid plaque) domain. We also noted that some variants have lower aggregation scores than WT α-SYN: T72K, T75K, A76E, and A78D. Of particular interest is S129Y α-SYN as phosphorylation of S129 is related to the facilitation of Lewy body (LB) formation and accelerated neurodegeneration in PD [22, 25]. To detect these predicted α-SYN TM variants from human post-mortem brain samples with sensitivity as well as high specificity, we used RNaseH2 PCR (rhPCR). First, we validated the specificity of rhPrimer sets using the template DNA containing mutations (S42Y, A53E) and WT α-SYN. Each rhPrimer set showed high specificity for a cognate mutation in an RNaseH2 dose-dependent manner (Supplementary Fig. 2a,b). rhPrimer sets for both TM variants and the corresponding WT were designed and tested (Supplementary Fig. 2c). Seven α-SYN TM variants namely L38I, S42Y, H50N, A53E, T72K, T75K, and S129Y were selected for rhPCR analysis of post-mortem midbrain samples of PD and age-matched controls. The rationale for selecting these seven hypothetical mutations was discussed in detail in the previous review [4]. The rhPCR of all the selected mutations indicated significantly higher loads of TM-generated α-SYN mutants in the PD cohort compared to control samples. The percentage load of total α-SYN TM variants in control samples was 6.7% whereas it accounted for 25.4% in PD. The incidence of TM was significantly higher in PD than in the control. There is a significant relationship between PD and TM incidence (χ^2^ = 14.186, *p* < 0.001) PD was more likely to have TM than control. (Fig. 2c). Individual analysis of the mutants showed a varied distribution ranging from 0% to 60.0%. In the control samples, the percentage load of each mutant analyzed was L38I (6.25%), S42Y (18.75%), H50N (0%), A53E (12.5%), T72K (0%), T75K (0%) and S129Y (6.25%). In the PD cohort, it was L38I (0%), S42Y (60.0%), H50N (20.0%), A53E (55.0%), T72K (15.0%), T75K (0%,) and S129Y (15.0%), respectively (Fig. 2d). Comparing sequences containing the target region between mRNA and the genomic DNA of the same subject confirmed that the mutations (S42Y and A53E) were generated by the TM event only in RNA, but not in the genomic DNA (Supplementary Fig. 3a,b). The significant increases in C-to-A transversion in α-SYN mRNA were confirmed using an α-SYN coding region-specific amplicon-sequencing. cDNA was extracted from 5 controls and 8 PD midbrain samples and genomic DNA was prepared from 4 controls and 3 PD samples of the same regions followed by PCR amplification for NGS sequencing. Among 12 possible base changes, we observed 4 significant base changes in both control and PD cDNA samples including A-to-C, T-to-G, G-to-T, and C-to-A (8-oxodG-dependent TM) with no such changes in genomic DNA. Interestingly, we observed the base changes of A-to-C and T-to-G in both controls and PD cDNA samples, but no significant differences between groups. However, G-to-T and C-to-A changes were observed in PD samples with significant differences in C-to-A, supporting our hypothesis (Supplementary Fig. 2d). Together, the results indicate that 8-oxodG-mediated TM event surely happens at a significantly higher rate in PD compared to controls, which generates various α-SYN TM mutants in the SN.

### S42Y α-SYN-positive LBs in the SNpc of PD and DLB brain

We next investigated whether α-SYN TM variants contribute to LB formation during PD pathogenesis. We developed a specific antibody against S42Y mutant as it has the highest aggregation score and is most abundantly found in PD among the analyzed α-SYN TM variants (Fig. 2b,d). Highly specific anti-S42Y α-SYN antibody was initially selected from four different animals (Supplementary Fig. 4a). This polyclonal rabbit anti-S42Y α-SYN antibody was thoroughly validated for selective detection of S42Y over WT α-SYN using a western blot (Supplementary Fig. 4b,c) and the SiMPull assay that we recently developed [33] (Supplementary Fig. 4d,e). The specificity of the antibody was also confirmed in total protein lysates prepared from *SNCA* KO HEK293 cells (Supplementary Fig. 4f) transiently overexpressing both WT and S42Y with different ratios (Supplementary Fig. 4 g). Using this highly specific anti-S42Y α-SYN antibody, we performed immunostaining to investigate whether there are LBs containing S42Y α-SYN mutant in the SNpc of PD. Three serial sections of the same PD brain were stained with hematoxylin–eosin (HE), anti-ubiquitin immunostaining to visualize LBs, and anti-S42Y antibody to determine if any of the detected LBs visualized by HE and anti-ubiquitin immunostaining contain S42Y α-SYN mutant. Several mature LBs that were positively stained for both HE and ubiquitin were also stained with anti-S42Y antibody (Fig. 2e, Supplementary Fig. 5a). Not all LBs were, however, positively stained for S42Y α-SYN, indicating the lower abundance of α-SYN TM variants. The percentage of LBs containing S42Y α-SYN was measured by double immunostaining for total (pink) and S42Y α-SYN (brown) in the SNpc sections from eight PD patients (Fig. 2f). We observed that each individual had a different number of total LBs and S42Y-positive LBs, and the average percentage of LBs containing S42Y was about 25.1% in the regions and sections analyzed (Fig. 2g,h). This observation was further supported by the detection of S42Y-positive LBs in DLB samples, which emphasizes the contribution of TM to the pathogenesis of α-synucleinopathy (Supplementary Fig. 5b). Taken together, these data strongly suggest that various α-SYN TM variants including S42Y which are generated by increased genomic 8-oxodG in PD might contribute to LB formation and PD pathogenesis.

### Significantly higher aggregation propensity of S42Y over WT α-SYN

**Fig. 3:**
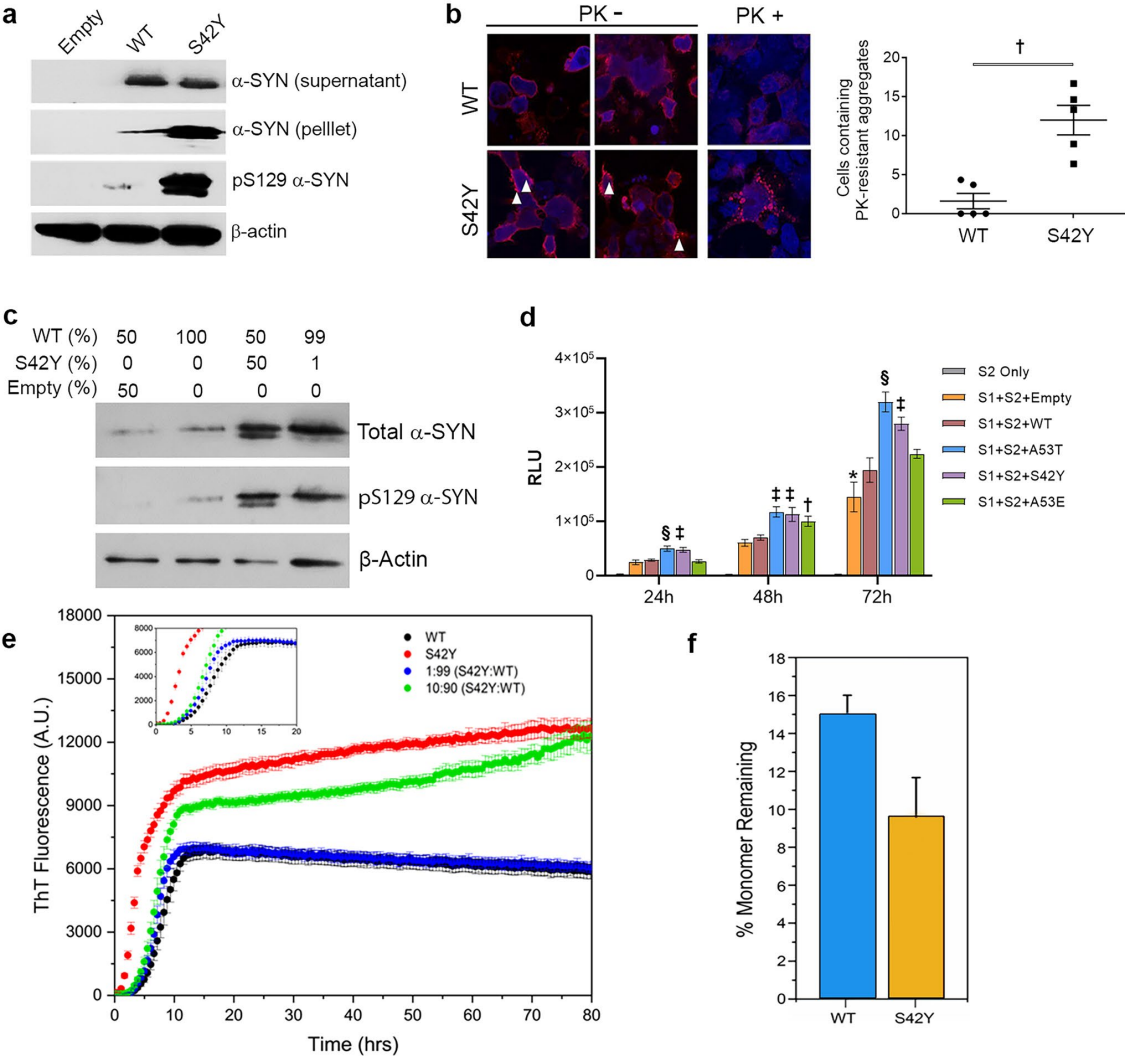
S42Y α-SYN exhibits stronger aggregation over WT and accelerates aggregation of WT α-SYN protein. **a** Western blot showing increased aggregation of S42Y α-SYN over WT. Triton X-100-soluble supernatant or –insoluble pellet prepared from *SNCA* KO HEK293 cells transiently transfected with either backbone vector (Empty), WT or S42Y α-SYN were analyzed using anti-α-SYN antibody. The insoluble fraction was further analyzed using anti-pS129 α-SYN antibody, confirming stronger aggregation of S42Y. β-actin in the soluble fraction was used as an internal control. **b** Overexpression of S42Y in *SNCA* KO HEK293 cells showed significant increases in cells containing PK-resistant aggregates compared to the WT overexpression. White arrow heads, perinuclear punctate α-SYN aggregates detected with α-SYN immunofluorescence staining. Data represent mean ± SEM. ‡*p* < 0.001 (Non-parametric t-test with Mann–Whitney post-hoc corrections, two-tailed p-values). **c** Western blot showing that overexpression of S42Y accelerates WT α-SYN aggregation. *SNCA* KO HEK293 cells were transfected with WT and S42Y α-SYN plasmids with various ratios, and Triton X-100 insoluble fractions were analyzed using anti-α-SYN or anti-S42Y antibodies. β-actin in the soluble fraction was used as an internal control. **d** Split luciferase complementation assay exhibits a small amount of S42Y accelerates WT α-SYN aggregation. *SNCA* KO HEK293 cells were transfected with split luciferase tagged with WT-α-SYN (S1, S2) together with various α-SYN constructs including WT, S42Y, A53T and A53E for 24, 48, and 72 h. α-SYN aggregations were assessed by luciferase activity. Data represent mean ± SEM. * *p* < 0.05, †*p* < 0.01, ‡*p* < 0.001, §*p* < 0.0001 (One-way ANOVA with Tukey’s multiple comparison test for each time point). **e** ThT fluorescence traces for α-SYN fibril formation for WT, S42Y, 1:99 S42Y:WT and 10:90 S42Y:WT. The mixed monomer sample ratios represent molar equivalents. For clarity, the inset plot shows the early ThT fluorescence traces up to 20 h. The total protein concentration for each experiment was 70 μM and was conducted at a pH = 7.4 at 37 °C with shaking. Traces shown are representative of at least 3 replicates each, and error bars represent the standard error of the mean (SEM). **f** BCA assay results showing the amount of monomer remaining at the endpoint of the 70 μM ThT assay. Residual monomer concentrations are shown as the percentage of the starting monomer concentration (14.99% for WT; 9.63% for S42Y). Error bars represent standard deviation

Next, we investigated the aggregation propensity of S42Y α-SYN in comparison to WT when it is expressed in cells. To get rid of the confounding effect caused by endogenous α-SYN, we established *SNCA* KO HEK293 cells using CRISPR/Cas9-mediated genome editing (Supplementary Fig. 4f). Following overexpression of either WT or S42Y, Triton X-100-soluble supernatant or -insoluble pellet was analyzed by a western blot, showing that a higher level of S42Y was detected in the insoluble fraction compared to WT α-SYN (Fig. 3a). We also observed increased phosphorylated S129 α-SYN (pS129) immunostaining, a widely used indicator for the α-SYN aggregation and facilitation of LB formation (Fig. 3a) [2, 22, 25]. α-SYN immunostaining after digestion with proteinase K (PK) demonstrated that the number of PK-resistant perinuclear punctae was also significantly increased in S42Y overexpressed cells, faithfully corroborating this finding (*p* = 0.0079) (Fig. 3b). Stronger aggregation propensity of S42Y over WT α-SYN urged us to investigate conformation of S24Y recombinant fibrils in comparison to WT α-SYN. Using cryo-electron tomography (cryo-ET), the morphological features of WT and S42Y α-SYN fibrils were analyzed. Tomograms of the S42Y fibrils revealed considerable conformational heterogeneity compared to WT fibrils (Supplementary Fig. 6a,b). The wild-type fibrils are predominantly present in the rod-like conformation with diameters varying from approximately 10 nm to 12 nm (Supplementary Fig. 6a). Fibrils from the S42Y mutant exhibited a continuum of polymorphism with a larger diameter range of 7 nm-14 nm (Supplementary Fig. 6b–h). This structural polymorphism is consistent with previous studies [26, 27, 42]. To investigate the structural profile of the S42Y α-SYN fibrils, we extracted individual fibril subtomograms and identified distinct types of structures as dominant conformers, ranging from the rod-like conformation similar to that observed in WT fibrils to polymorphs of twisted fibrils (Supplementary Fig. 6c–h). These polymorphs have a distinctly different pitch, defined as the length of a 360° helical turn of the fibril, ranging from 68 to 92 nm. The most predominant mutant fibril species is characterized by a twisted morphology with a pitch range of ~ 72 nm. This suggests that different polymorphs may exhibit differential biochemical properties. Overall, the result confirmed S42Y α-SYN to have a higher potential to aggregate compared to WT α-SYN, which strongly fortifies the premise to study the effect of this mutation in the aggregation of the WT parental α-SYN.

### S42Y mutant accelerates WT α-SYN aggregation

We hypothesized that a small number of α-SYN variants with altered aggregation characteristics generated by TM might accelerate the aggregation of WT α-SYN. Using both the cell-based analysis and the aggregation assay using recombinant proteins, we sought to test this hypothesis. After transfection of *SNCA* KO HEK293 cells with different ratios of WT to S42Y, total protein lysates were analyzed for S42Y and total α-SYN, showing that S42Y protein could be faithfully controlled to desired levels without affecting total α-SYN levels (Supplementary Fig. 4 g). Two different ratios of S42Y to WT α-SYN (50:50 and 1:99) were tested to see if small additions of S42Y could modify WT aggregation. Triton X-100 insoluble fraction was immunoblotted for total α-SYN and pS129, demonstrating that both 1% and 50% of S42Y expressions significantly enhanced the aggregation of WT α-SYN (Fig. 3c). This finding was corroborated by a split-luciferase complementation assay which showed a significant increase in luciferase activity (as a measure of aggregation) in the presence of 1% S42Y compared to WT only over a period of 72 h (*p* = 0.0003 at 24 h; *p* = 0.0004 at 48 h; *p* = 0.0006 at 72 h) (Fig. 3d). While adding 1% A53T to WT similarly accelerated WT aggregation as S42Y did, A53E failed to increase WT aggregation. The kinetics of fibril formation of WT α-SYN alone, S42Y mutant alone, and WT α-SYN in the presence of small molar quantities (1% and 10%) of S42Y mutant were investigated by Thioflavin (ThT) fluorescence (Fig. 3e). Kinetics of aggregation of S42Y are faster than WT alone consistent with cell-based assay. The shape of the aggregation curves and final ThT intensities are different, suggesting a different mechanism of aggregation, different binding affinities of ThT to the WT versus S42Y fibrils, or increased fibril formation. Experiments performed at lower concentrations of WT α-SYN show similar trends in aggregation kinetics (Supplementary Fig. 7). In the presence of small molar quantities of monomer S42Y mixed with monomer WT α-SYN (1:99 and 10:90, S42Y:WT molar equivalents), fibril formation was slightly more rapid, in a dose-dependent manner, relative to WT alone. The inset in Fig. 3e highlights the early time points of ThT fibril formation and shows that the macroscopic lag phase is affected in S42Y relative to WT. Monomer depletion experiments (Fig. 3f) indicate greater depletion of monomer for S42Y relative to WT α-SYN consistent with the higher ThT values observed for S42Y in the mature fibril stage of the kinetics experiments and cell-based assays.

### S42Y α-SYN mutant accelerates neuronal degeneration

**Fig. 4:**
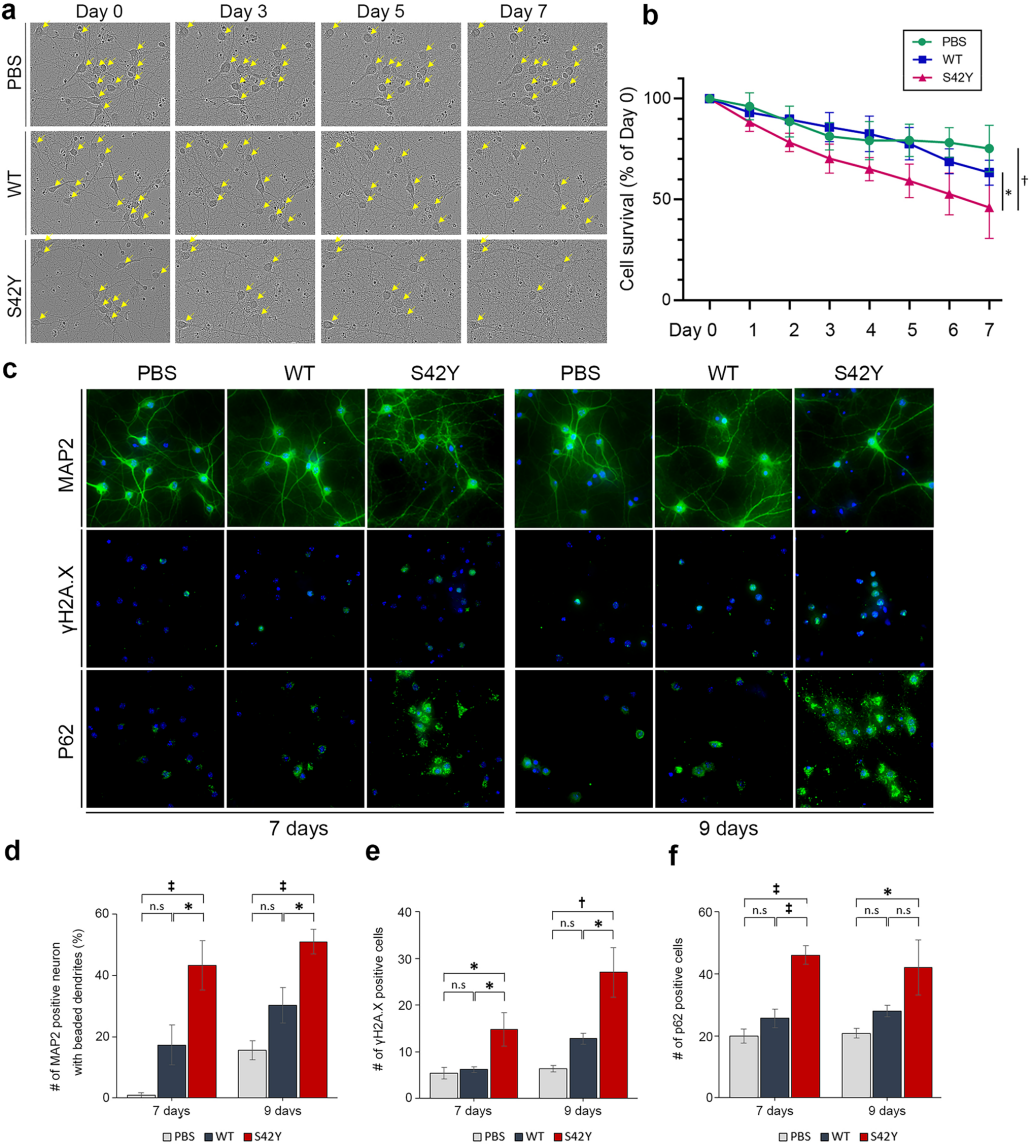
S42Y α-SYN fibrils show increased neurotoxicity compared to WT. Primary mouse cortical neuron cultures were incubated with either WT or S42Y fibrils over time. **a** Time-course neuronal degeneration was assessed using serial phase-contrast images for 7 days. Yellow arrowheads indicate intact cell bodies. **b** S42Y α-SYN fibrils resulted in significant neuronal death over a period of 7 days compared to WT fibrils or PBS. Data represent mean ± SEM. (Two-way repeated measures ANOVA to analyze the cell survival (%) for 7 days (F7, 14 = 4.163, *p* = 0.007) followed by Tukey’s post hoc tests. S42Y reduced the cell survival (%) compared to PBS treatment (*p* = 0.010) and WT treatment (*p* = 0.024) **c**–**f** S42Y α-SYN fibrils increased neuritic degeneration, double-strand DNA damage, and autophagic cell death. Representative confocal fluorescence micrographs of MAP2, γH2AX, and p62 in primary mouse cortical neurons treated with PBS, WT, or S42Y α-SYN fibrils for 7 days or 9 days (**c**). The number of MAP2-positive neurons having “beaded” neurites (**d**), γH2A.X-positive (**e**), and p62-positive neurons (**f**) was counted. Data represent mean ± SEM. *p < 0.05, †*p* < 0.01, ‡*p* < 0.001, *n.s.* non-significant (One-way ANOVA with Tukey’s multiple comparisons test for each time point of MAP2, rH2A.X, and P62 between three groups)

**Fig. 5:**
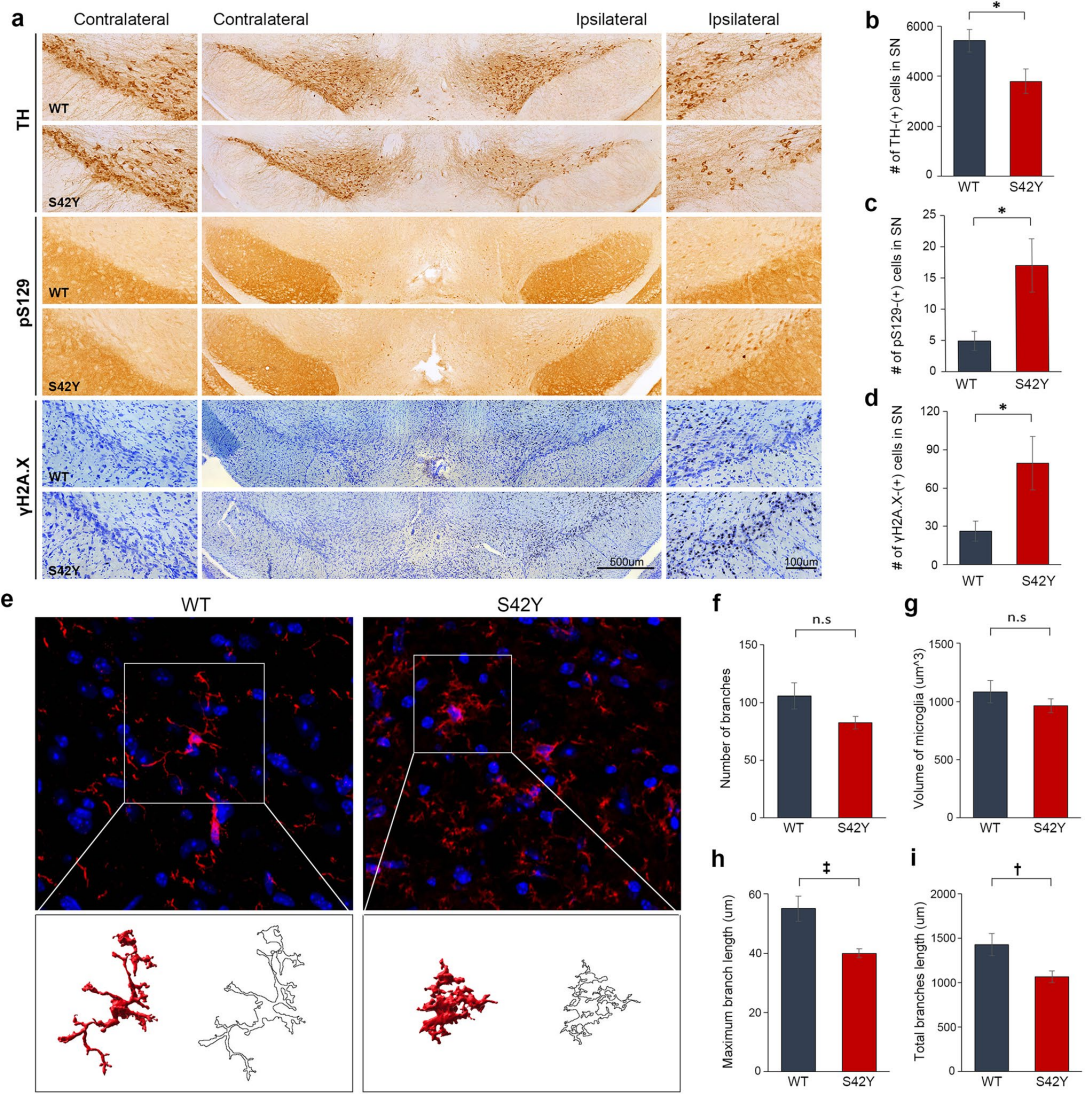
Overexpression of S42Y α-SYN in the mouse SNpc shows accelerated degeneration of dopaminergic neurons compared to WT. AAV containing WT or S42T was unilaterally injected into the SN, and mice were sacrificed after 14 days. **a** Representative photomicrographs of immunostainings for TH, pS129, and γH2A.X in the ipsilateral and contralateral SN. **b**–**d** The number of TH-positive (**b**), pS129-positive (**c**), and γH2A.X-positive neurons (**d**) were counted in the WT and S42Y α-SYN overexpressed SN. **e**–**i** Microglia are highly activated by S42Y compared to WT α-SYN. Morphometric assessment of microglia was performed after immunolabeling with Iba-1 (red) and nuclear DAPI staining (blue) (**e**). The number of branches (**f**), volume (**g**), the maximum length of branches (**h**), and total length of branches (**i**) were measured in the WT and S42Y α-SYN overexpressed SN. Data represent mean ± SEM. *p < 0.05, †*p* < 0.01, ‡*p* < 0.001, *n.s.* non-significant (Non-parametric *t*-test with Mann–Whitney post-hoc corrections, two-tailed *p*-values)

It has been well documented that α-SYN is a toxic protein that increases the rate of neuronal death in its mutant forms or under conditions where its expression levels are elevated [14, 80]. Exogenous added α-SYN pre-formed fibrils (PFF) can also seed the formation of LB-like intracellular inclusions in cultured cells [48]. To investigate S42Y TM variant-induced neuronal death compared to WT α-SYN, we first treated mouse primary cortical neuronal culture with the pre-formed fibrils (PFF) prepared from S42Y or WT α-SYN recombinant proteins and performed the time-course morphological analysis over 7 days (Fig. 4a). Treatment with S42Y PFF significantly increased neuronal death compared to WT (*p* = 0.024) or PBS treated groups (*p* = 0.010) (Fig. 4b). Neuronal degeneration was further investigated by tracking neuritic beading after MAP2 immunostaining (Fig. 4c upper panel, Supplementary Fig. 8a) [36, 57]. Neurite beading was significantly increased in neurons treated with S42Y PFF compared to other conditions (*p* < 0.05 between WT vs S42Y at 7 and 9 days) (Fig. 4c,d). It has been shown that oxidized and misfolded α-SYN causes DNA strand breaks and DNA-damage response (DDR) followed by increased neuronal death in both in vitro and in vivo conditions [51, 75]. We investigated γH2A.X, a well-established marker for DNA strand breaks, and observed significant increases in γH2A.X levels in cells treated with S42Y PFF both at 7 and 9 days following treatments (*p* < 0.05 between WT vs S42Y at 7 and 9 days) (Fig. 4c,e). LB-like α-SYN inclusions in cells formed by introducing α-SYN fibrils trigger activation of the p62-mediated autophagy-lysosomal pathway [78]. Primary cortical neurons treated with S42Y PFF significantly increased the p62 signal, suggesting autophagolysosomal overloads (*p* < 0.001 between WT vs S42Y at 7 days) (Fig. 4c,f). We further investigated the role of S42Y α-SYN in nigral dopaminergic neuronal degeneration by adeno-associated virus (AAV)-mediated overexpression of S42Y or WT α-SYN in the SN. Overexpressed S42Y mutant protein in the SN was visualized by an anti-S42Y antibody (Supplementary Fig. 8b). Animals were sacrificed 14 days after injection to see acute effects exerted by S42Y in comparison to WT. WT α-SYN, as expected, did not cause any significant pathological changes in the SN in such a short period of time, while S42Y induced significant dopaminergic neuronal loss (*p* < 0.05) (Fig. 5a,b), elevated pS129 immunostaining (*p* < 0.05) (Fig. 5a,c), and increase in γH2A.X levels (*p* < 0.05) (Fig. 5a,d). AAV-mediated overexpression of human α-SYN in the SN exhibited microglial activation over the course of dopaminergic neuronal degeneration [71]. We performed a morphometric analysis of microglial activation, showing that nigral overexpression of the S42Y mutant significantly increased activated microglia with decreases in the length of process branches (Fig. 5e–i). Taken together these results suggest that, compared to WT α-SYN, the S42Y mutant exhibits significantly stronger neurotoxicity.

## Discussion

In the present study, we investigate 8-oxodG-mediated TM as a novel mechanism contributing to α-SYN aggregation in the pathogenesis of sporadic PD. Increased oxidative stress has been a primary factor contributing to the aggregation of α-SYN and the pathogenesis of Parkinson’s disease [45, 63, 66]. Previous studies showed that oxidative DNA lesions, such as 8-oxodG, accumulate in genomic and mitochondrial DNA in both animal models and post-mortem PD brains [1, 23, 54]. 8-oxodG is a non-bulky lesion that does not stall RNA pol II to induce an OGG1-mediated base excision repair mechanism [39]. An in vitro study demonstrated that about 10–15% of mutagenic adenine-inserted mRNA could be generated as RNA pol II reads a DNA template containing 8-oxodG [6]. Although the accumulation of 8-oxodG in DNA has been implicated in several diseases, such as cancer, neurodegeneration, and cardiovascular disease [5], no studies have investigated whether or not 8-oxodG directly contributes to the pathogenesis of these diseases.

Our current study presents compelling data showing that increased accumulation of 8-oxodG leads to the generation of novel mutants of α-SYN through TM, which contributes to PD pathogenesis by promoting α-SYN aggregation and increasing neuronal toxicity. First, we confirmed elevated levels of 8-oxodG in genomic DNA from the SN of PD compared to the age-matched controls, which was previously observed by Alam et al. [1]. In addition to dopaminergic neurons, other types of cells also exhibited increased 8-oxodG staining. It will be interesting to investigate cell-type-specific roles of 8-oxodG-derived TM in the pathogenesis of other synucleinopathies including dementia with Lewy bodies (DLB) and multiple system atrophy (MSA). While it has been shown that regions with a high frequency of recombination and single nucleotide polymorphisms (SNPs) were located within chromosomal regions with high 8-oxodG [55], no reports have supported the biased distribution of 8-oxodG towards specific genes, which implies that the TM event can affect a multitude of functional genes. We focused on investigating, however, the effects of TM event with respect to α-SYN aggregation and PD because the generation of α-SYN TM variants with high aggregation potential can potentially lead to template-directed misfolding of the parent WT protein in a nucleation-dependent manner [13, 79]. Target-specific analysis of 8-oxodG on the *SNCA* coding region also revealed a significant elevation in the PD brain, which served as a foundation for testing the generation of TM-mediated α-SYN mutants. We failed to observe differences in total OGG1 levels or its 8-oxodG repairing activity between PD and control conditions, suggesting that 8-oxodG accumulation might reach beyond the repair capacity of OGG1 in PD. A previous study demonstrated that OGG1-2a, a mitochondrial isoform, is elevated in the SN of PD [23]. But this specific isoform lacks enzymatic activity [24]. Together, the results suggest that 8-oxodG accumulation in PD results from oxidative stress-mediated DNA damage build-up and not from the deficit or failure of OGG1-mediated repair machinery.

It was predicted that a total of 43 missense mutations can be generated by the 8-oxodG-mediated TM event [4]. Due to technical limitations, it was challenging to determine all TM-derived α-SYN mRNA species out of WT using unbiased techniques due to their low abundance and probability of occurrence compared to WT mRNA, which prompted us to develop a target-specific method, RNaseH2 PCR. A potential caveat of this technique was the use of total RNA lysates, which limited the interpretation of cell-type-specific contributions of the selected TM mutations. We focused on seven positions distributed throughout the length of the *SNCA* coding region that are categorized into: (i) higher TANGO aggregation scores than WT α-SYN (S42Y, H50N, L38I); (ii) the mutation reported in a familial PD (A53E); (iii) a key amino acid for phosphorylation-mediated aggregation (S129Y); (iv) mutations within the NAC-domain (T72K and T75K). We observed an overall higher load of TM mutants in PD compared to age-matched controls. It has been demonstrated that the sequence context of DNA around the 8-oxodG can influence the TM event [6], suggesting potential hotspots for the TM event. Although our study does not provide concrete evidence of a biased generation of each TM variant from the *SNCA*, it was interesting to note that some mutants (S42Y, A53E) were observed more frequently compared to other ones (T75K). As expected, α-SYN TM variants were also detected in age-matched control brains. Age-dependent oxidative DNA damage and its effects on gene regulation were previously reported [47]. The Baltimore Longitudinal Study of Aging (BLSA) showed that LBs are also detected in the normal aging cohort (incidental LBs), although their rate of incidence was significantly lower (8.3%) compared to PD (100%) [34, 50]. This is in line with our observation of an overall higher load of TM mutants in PD compared to age-matched controls. It will be important to investigate whether the aging process contributes to the generation of α-SYN TM variants by investigating a young cohort.

Next, we studied the contribution of TM-generated α-SYN mutants on the aggregation of WT α-SYN and the formation of LBs. Due to technical limitations to evaluate the cumulative effects of total α-SYN TM variants on the overall aggregation of α-SYN, we focused on the pathologic contribution of TM with respect to S42Y α-SYN. The in-silico aggregation analysis indicated that S42Y has the highest aggregation propensity, and the S42Y transcript was most frequently detected in the PD midbrain among all tested α-SYN TM variants. Indeed, recently Ulamec et al. showed that alanine substitution at S42 abolishes all aggregative properties of the monomer [73]. We developed a highly specific polyclonal antibody for S42Y α-SYN after a negative screening against WT α-SYN. The specificity of the antibody was well validated using several biochemical and microscopy-based assessments, demonstrating that it detects S42Y but not WT α-SYN at a specific concentration. Using this antibody, we were able to detect LBs stained with S42Y in the PD brains. This is the first evidence proving that TM-generated α-SYN mutants could play roles in LB formation in PD pathogenesis. As expected, however, the number of LBs positively stained with S42Y were significantly less among total LBs. Considering that more than 40 α-SYN TM variants could be generated individually or in random combinations, the collective effects of TM on α-SYN aggregation and PD pathogenesis could be substantial. In accordance with the prediction by the TANGO algorithm, we observed significantly higher aggregation of S42Y α-SYN compared to WT α-SYN in various experimental conditions.

Cryo-ET revealed that the S42Y fibrils exhibited considerable conformational heterogeneity compared to WT fibrils. The polymorphs of WT full-length α-SYN fibrils were previously reported [42]. This structural heterogeneity of the S42Y fibrils may explain its increased aggregation propensity. Since it was technically not feasible to obtain the complete profile of all α-SYN TM variants, we could not have an accurate estimation of the relative levels of the α-SYN mutants with respect to the WT. However, to examine the hypothesis that small amounts of α-SYN mutant with strong aggregation potential can accelerate WT α-SYN aggregation, we tested the α-SYN aggregation in the conditions where 1% S42Y α-SYN was present with the 99% WT compared to the only WT α-SYN using recombinant proteins as well as controlled expression in the cells. Both conditions similarly showed that small addition of S42Y to large amounts of WT α-SYN, in fact, accelerates aggregation kinetics. These data demonstrate how 8-oxodG-derived TM event could contribute to α-SYN aggregation and LB formation despite the condition that only small amounts of α-SYN TM variants are generated and added to WT. It has been shown that the region comprising residues 36–42 in the N-terminal region of the α-SYN monomer is important in controlling initial dimer contacts and aggregation kinetics into mature fibrils [19, 32]. Further, it has been shown that altering the types of residues within this region referred to as the P1 region can dramatically change the kinetics of aggregation, with Buratti et.al. having shown that aromaticity at Y39 is essential for aggregation [7]. Our work here showing the ability of S42Y to accelerate the aggregation of α-SYN further underscores the central importance of S42 in the so-called “master regulator” region [73]. Additionally, this study demonstrated that L38I α-SYN rapidly forms amyloid fibril. While we failed to detect L38I by our PCR-based approach in the screened cohort, L38I α-SYN is one of the predicted α-SYN TM variants with a higher TANGO aggregation score than WT. In-silico analysis also predicted generation of mutants with lower aggregation scores compared to WT α-SYN. It is necessary to further investigate how the small increases in mutants α-SYN with various aggregation propensities in the same cells would affect its overall aggregation.

Finally, we examined the neurotoxicity of S42Y α-SYN in comparison with WT in the primary neuronal culture condition and in the AAV-mediated α-SYN overexpression model in mice. Accumulating evidence indicates that increased aggregation of α-SYN exerts pronounced neurotoxic effects by compromising many intracellular as well extracellular targets [14, 17, 18, 30, 59]. Our study also demonstrated that either in vivo overexpression of S42Y or the addition of S42Y PFF to the primary neuronal culture resulted in rapid neuronal death compared to WT conditions. This might be attributed to the rapid oligomerization kinetics of and toxicity of S42Y α-SYN.

Taken together, this study has shown 8-oxodG-mediated TM event to be a novel mechanism that underlies aggregation of α-SYN by the generation of new mutant species. The majority of idiopathic PD cases are of the late-onset, sporadic form with cytoplasmic α-SYN aggregates, which suggests that increasing levels of aggregation and toxicity do not depend only on genetic mutations in *SNCA*. However, to date, studies attempting to elucidate the molecular mechanism of α-SYN aggregation have focused solely on the biochemical properties of mutant protein species found in rare familial forms of PD. By contrast, we show in this study that accumulated 8-oxodG lesions in the protein-coding area of the *SNCA* gene in PD could generate a variety of α-SYN TM variants and contribute to LB formation by potentially accelerating the aggregation process*.*

Currently, over 100 oxidative DNA adducts have been identified, ranging from those with modifications of the bases (e.g., 8-oxodG, 8-oxodA, thymidine glycol, 5-hydroxylysine, and 5-hydroxyuracil) and nucleotides (abasic or cyclic forms; e.g., 2-deoxyribonolactone, 5′,8-cyclo-2’-deoxyguanosine, and 5′,8-cyclo-2’-deoxyadenosine) to those with breakage of the phosphate backbone [28, 49]. Whether all these DNA modifications contribute to a generation of α-SYN RNA mutants is yet to be elucidated. In addition to 8-oxodG-derived mutagenesis, other types of TM were also reported. It has been shown that GAGAG motifs in RNA are hot spots for a dinucleotide deletion (ΔGA) that leads to + 1 reading frame shift of amyloid precursor protein and ubiquitin-B in Alzheimer’s disease (AD) and Down syndrome [74]. The recent study also suggests that transcriptional fidelity could be compromised by various factors, leading to transcriptional error and disease pathogenesis such as AD [12]. Using total RNA or DNA prepared from the midbrain samples, we were able to detect various α-SYN TM species from RNA but failed to see amplification from genomic DNA. However, the possibility that some RNA variants might also come from somatic mutations cannot be completely excluded. Accumulating evidence indicates that somatic mutations in the brain can arise during brain development [35] and aging with brain-region specificity [44]. While somatic single nucleotide variants (SNVs) on *SNCA* have not been reported in PD [41, 43], somatic copy number gains of SNCA in nigral dopaminergic neurons of PD were reported [52]. As we exhibited with S42Y, a small proportion of these mutants might contribute to an acceleration of overall α-SYN aggregation. Although we demonstrated the significant impact that an event like TM can have on PD by studying one mutation in-depth, it can be well understood the broader impact that a combination of all the other possible mutants can have on α-SYN aggregation and pathogenesis of PD. We believe this study will not only represent a major advancement in our understanding of the pathogenesis of PD but will also provide additional information that might prove useful in the study of orphan diseases (e.g., amyotrophic lateral sclerosis and multiple system atrophy) and other synucleinopathy (e.g., Dementia with Lewy Bodies).

### Supplementary Information

Below is the link to the electronic supplementary material.

Supplementary file1 (DOCX 3846 KB)

Supplementary file2 (PDF 425 KB)

Supplementary file3 (PDF 62 KB)

Supplementary file4 (PDF 90 KB)

## Data Availability

Tomograms of WT and S42Y α-SYN fibrils have been deposited in the Electron Microscopy Data Bank under the accession codes EMD-28752 and EMD-28753, respectively.
