# Supplementary material for: Transcriptional mutagenesis of α-synuclein caused by DNA oxidation in Parkinson’s disease pathogenesis
Source: Acta Neuropathol. 2023 Sep 23;146(5):685–705. doi: 10.1007/s00401-023-02632-7 (PMC10564827; doi:10.1007/s00401-023-02632-7)
Supplement: Supplementary file 1 — Supplementary file1 (DOCX 3846 KB) [file 401_2023_2632_MOESM1_ESM.docx]

**
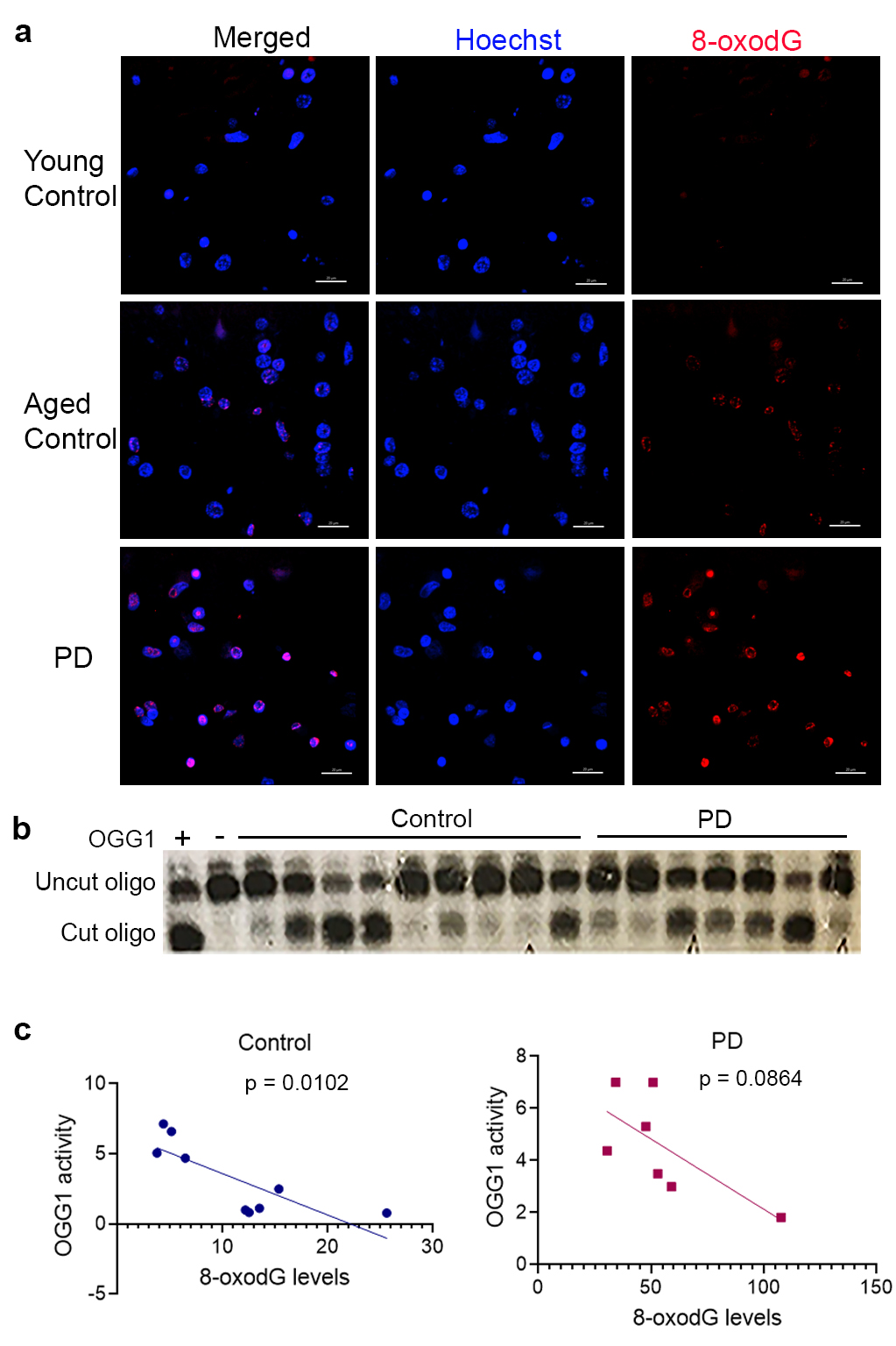
**

**Supplemental Fig. 1 a** Photomicrographs of 8-oxodG immunofluorescence stainings (red) in young and aged-matched controls and PD midbrain samples with nuclear hoechst stainings (blue). Scale bar = 20µm **b** Autoradiograph of OGG1 activity assay. Radiolabeled oligonucleotides containing 8-oxodG were cleaved by tissue homogenates prepared from control or PD midbrain samples followed by gel separation. **c** Controls showed a significant correlation between 8-oxodG levels and OGG1 activity (p=0.01; R^2^= 0.6356) while no significant correlation was observed in PD (p=0.06; R^2^=0.4747).


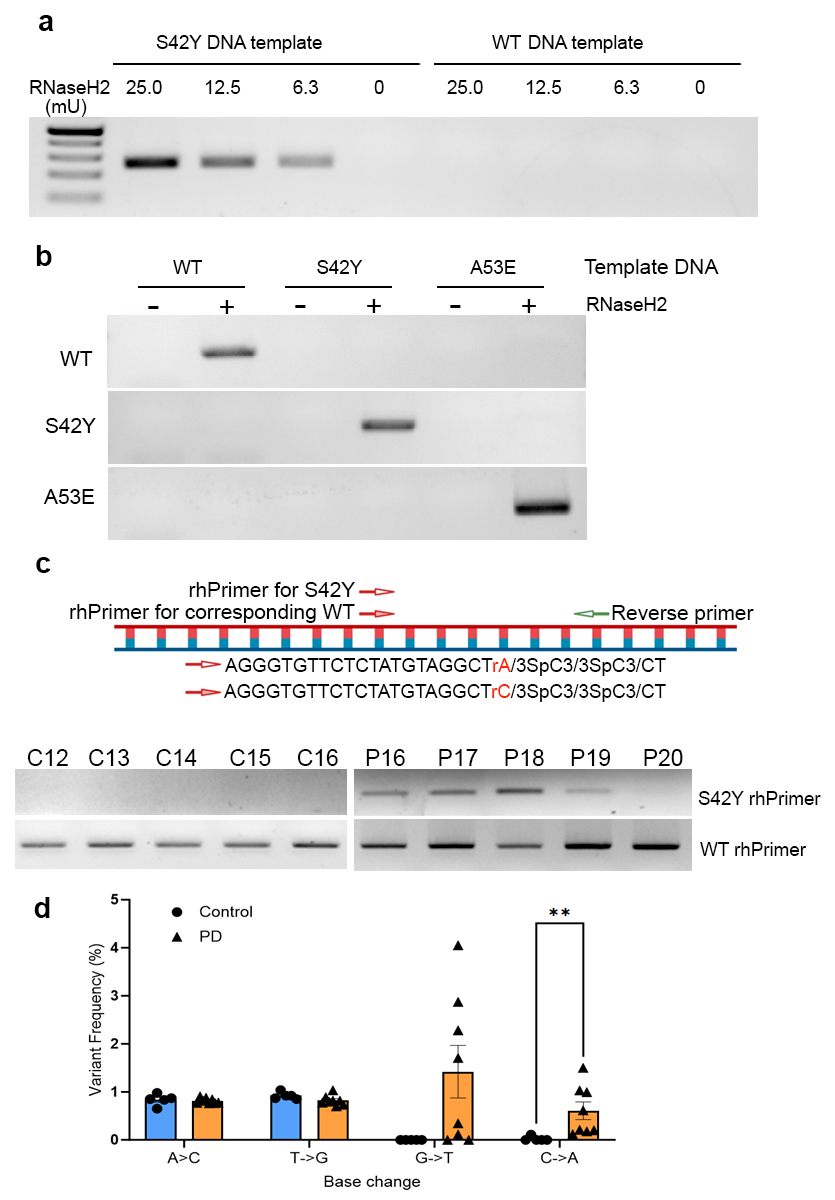


**Supplemental Fig. 2** Validation of the specificity of rhPCR. **a** RNase H2 concentration-dependent specificity of rhPCR. A primer set for S42Y α-SYN was tested against either WT or S42Y α-SYN plasmid with varying concentrations of RNase H2. **b** The specificity of each primer set for WT, S42Y, and A53E was tested using template DNA having each of α-SYN species. **c** Schematic representation of rhPrimer design to detect the TM mutants of α-SYN. rhPrimer sets for the corresponding WT were also designed. At the position of mutation, primers have a RNA base (red) forming a heteroduplex with the template cDNA, which is followed by blocking groups that prevent taq polymerase amplification until it is cleaved by RNase H2. cDNA prepared from 5 controls and 5 PD samples were amplified for S42Y α-SYN and a corresponding WT using RNase H2 PCR. The image was generated using Biorender. **d** Amplicon-seq analysis of the protein coding region of α-SYN cDNA in controls and PD midbrain samples. cDNA of 5 controls and 8 PD midbrain samples containing the substantia nigra was subject to a target-specific PCR amplification of an α-SYN coding region followed by NGS.


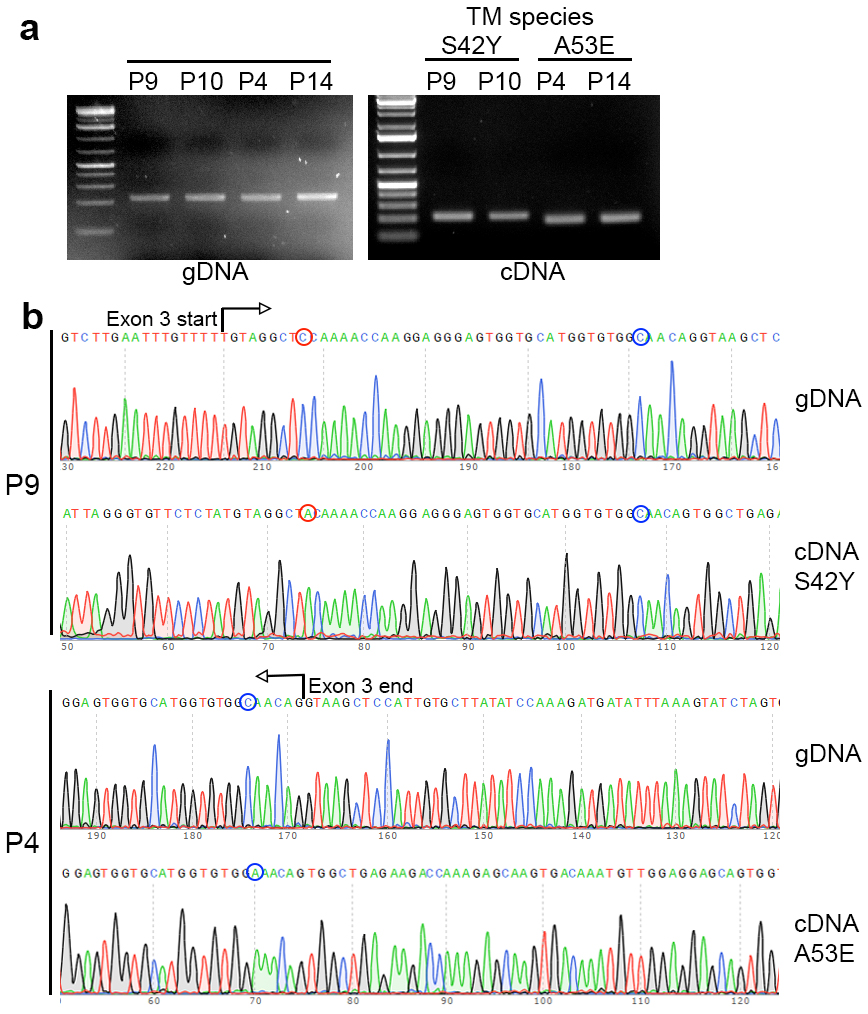


**Supplemental Fig. 3**  Sequence analysis of the amplified region from cDNA and genomic DNA (gDNA) from the same PD sample showed the presence of S42Y *SNCA* mutation (C🡪A) only in the cDNA but not in the gDNA. **a** Representative gel images of the *SNCA* regions containing both S42 and A53 for gDNA (left, a regular PCR) and for cDNA (right, RNaseH2 PCR). Following cloning into a sequencing plasmid, the amplicons were sent for a Sanger sequencing. **b** Representative chromatograms of sequencing of gDNA and cDNA. Nucleotides for TM positions are highlighted with circles: S42 (red circle) and A53 (blue circle).


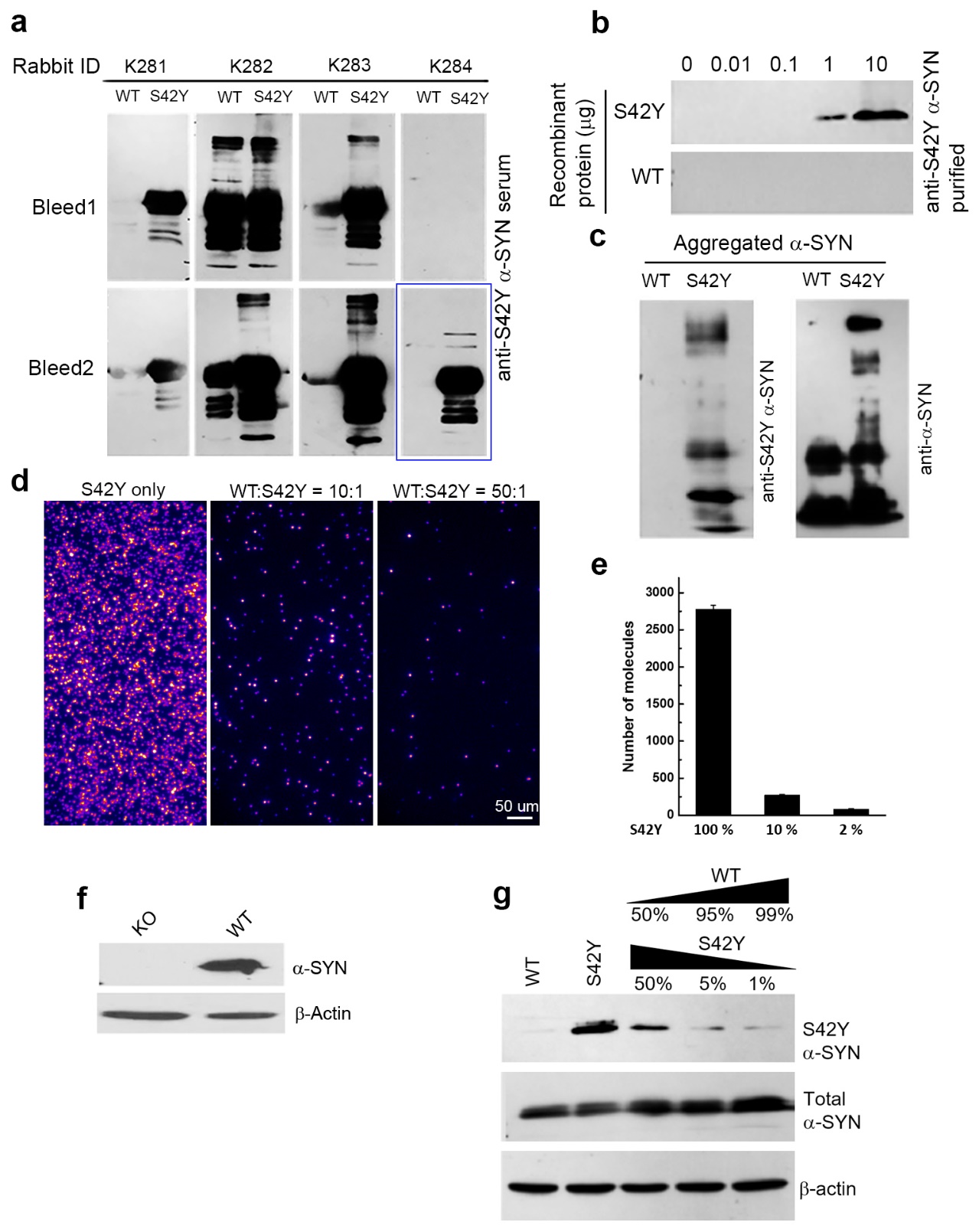


**Supplemental Fig. 4** Validation of the specificity of anti-S42Y antibody. **a** Western blot of recombinant WT and S42Y α-SYN with the first and second bleeds of anti-S42Y antibody extracted from four different animals (ID: K281, 282, 283, 284) demonstrated that K284 bleed 2 most selectively detected S42Y from WT αSYN (blue box). **b** Western blot image showing specificity of the anti-S42Y antibody to S42Y α-SYN when titrated with increasing amounts of S42Y and WT recombinant proteins (0, 10ng, 100ng, 1000ng and 10,000ng). **c** Western blot image showing the specificity of the anti-S42Y antibody to aggregated S42Y α-SYN without detecting any aggregated WT α-SYN (left panel) while anti-α-SYN antibody detected both WT and S42Y aggregates (right panel). **d** and **e** The specificity of anti-α-SYN antibody was confirmed using a SiMPull technique. Recombinant S42Y α-SYN was mixed with WT at different ratios (0, 2, 10%), and S42Y signals detected by anti-S42Y antibody were counted using a SiMPull, showing the expected proportional detections. **d** Representative single-molecule images of three independent experiments. **e** An average number of fluorescent spots of S42Y α-SYN molecules per imaging area. More than 20 images were taken and error bars denote standard deviation (s.d.). Scale bar, 5 µm. **f** Successful establishment of *SNCA* KO HEK293 cells was validated using western blot for α-SYN. **g** Western blot image showing the specificity of anti-S42Y antibody in total protein lysates prepared from *SNCA* KO HEK293 cell transfected with WT and S42Y at various ratios.

**
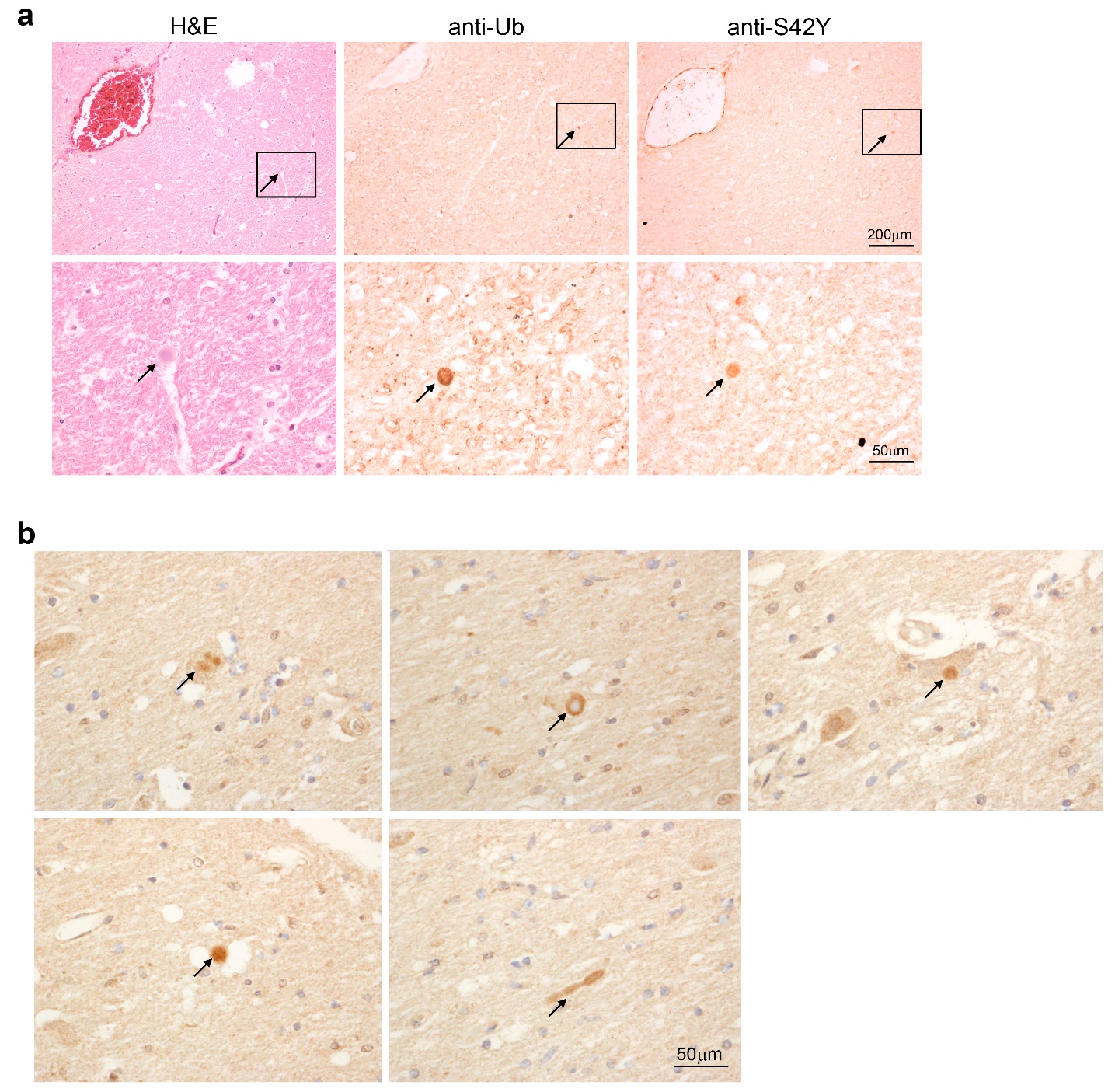
**

**Supplemental Fig. 5** Validation of the specificity of anti-S42Y antibody. **a** Representative photomicrographs showing a LB immuno-positive for S42Y α-SYN. LBs were visualized using H&E staining and anti-ubiquitin antibody together with anti-S42Y antibody in 6µm-apart serial sections of PD midbrain. Black arrows, co-labeled LB. Lower panels, magnified boxed areas in the upper panels. **b** Photomicrographs showing S42Y-positive LBs and LN (lower middle panel) in various postmortem brain samples of DLB. Black arrows indicate LBs positively stained with anti-S42Y antibody.

**
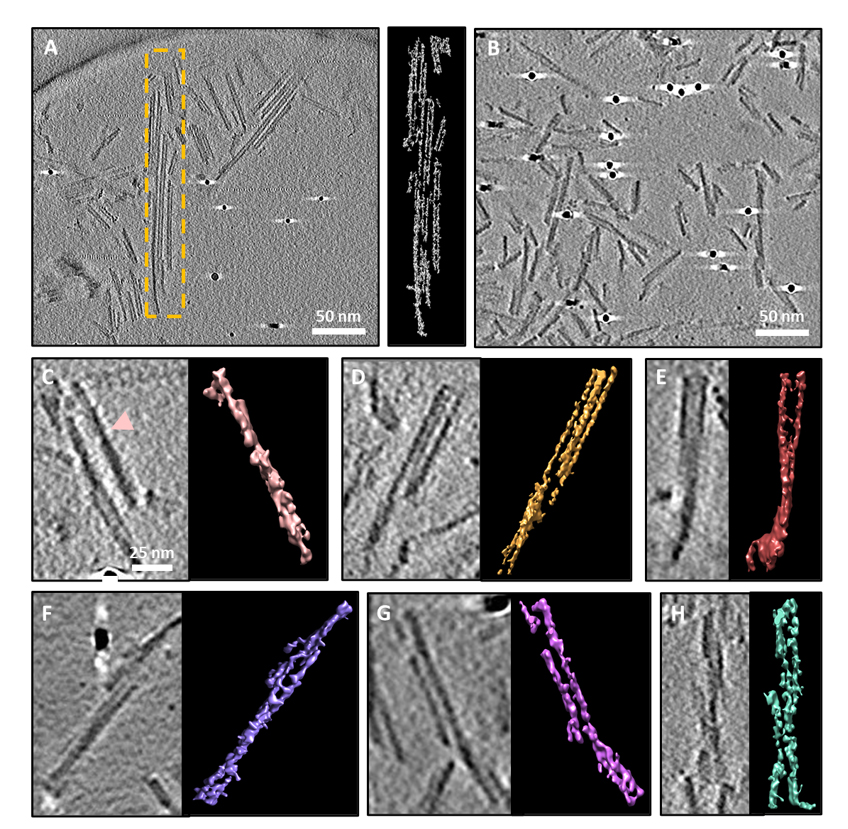
**

**Supplemental Fig. 6** Cryo-EM tomography analysis shows considerable conformational heterogeneity of S42Y α-SYN fibrils compared to WT fibrils. Representative tomogram of WT (A) and S42Y α-SYN fibrils (B). Middle panel presents magnified boxed area in panel A. Subtomograms of S42Y fibrils showing various structural polymorphisms (C-H).

**
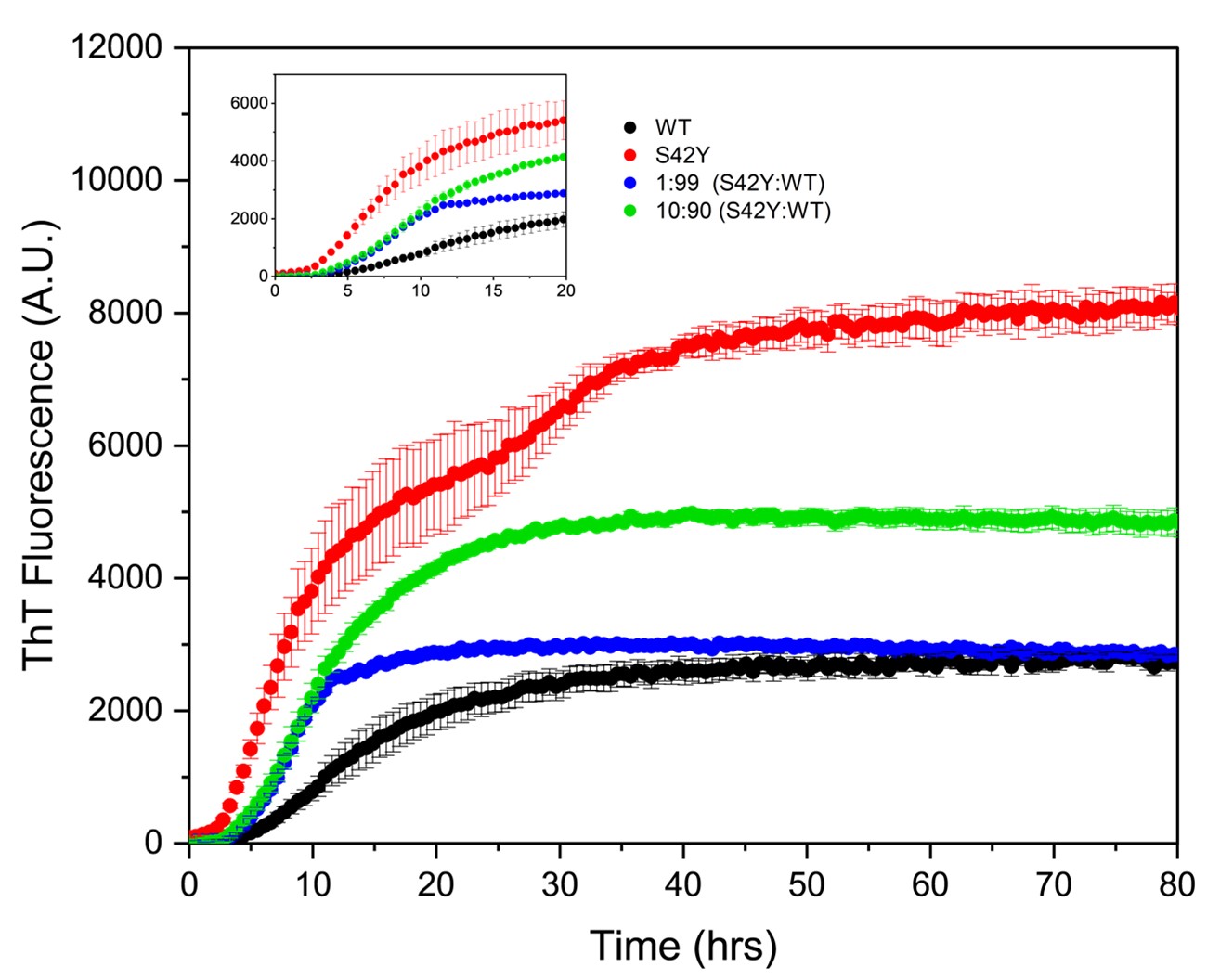
**

**Supplemental Fig. 7** ThT kinetic assay of WT and S42Y mutant monomers. Assays were conducted at 35μM monomer concentration, at 37°C, and pH=7.4 with shaking. Traces shown are representative of at least 3 replicates each, and error bars represent the standard error of the mean (SEM).

**
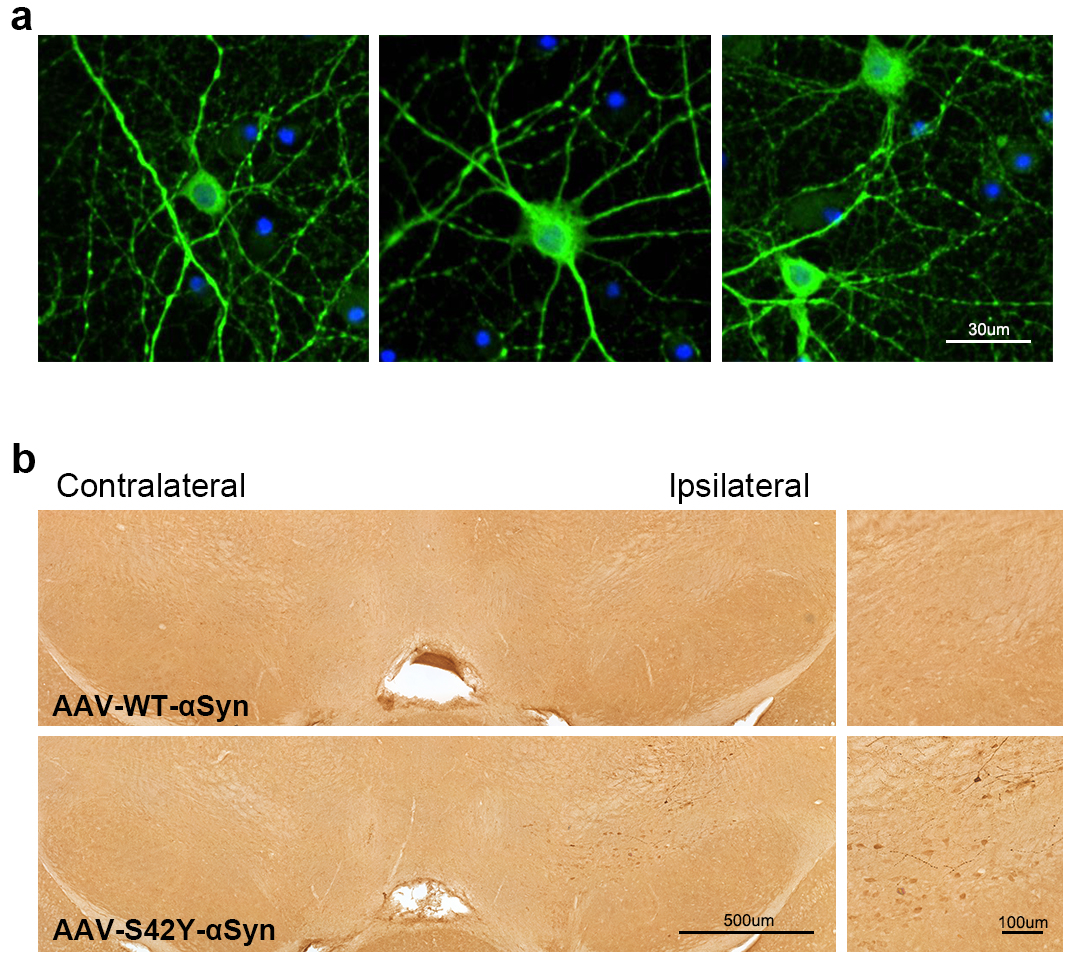
**

**Supplemental Fig. 8­ a** Representative fluorescent images of S42Y-induced neuritic beadings after immunostained with MAP2. **b** Anti-S42Y α-SYN antibody specifically detected overexpressed S42Y α-SYN in the SN but not WT α-SYN. The ipsilateral SNpc regions were magnified (right panel).
