# Supplementary material for: Transcriptional mutagenesis of α-synuclein caused by DNA oxidation in Parkinson’s disease pathogenesis
Source: Acta Neuropathol. 2023 Sep 23;146(5):685–705. doi: 10.1007/s00401-023-02632-7 (PMC10564827; doi:10.1007/s00401-023-02632-7)
Supplement: Supplementary file 2 — Supplementary file2 (PDF 425 KB) [file 401_2023_2632_MOESM2_ESM.pdf]

Sample ID	Repository ID	Diagnosis	Age	Sex	PMI (hours)	RIN	Repository	NFT Braak Stage	LB Braak Stage
C1	PDC022	Control	65	M	12	6.5	PD UK Brain Bank	Not available	Not available
C2	PDC035	Control	89	F	13	7.3	PD UK Brain Bank	Not available	Not available
C3	Hct ZZA_16_0001	Control	89	M	25.5	8.2	U of Miami, Brain Bank	I	No LBs
C4	Hct ZZR_16_0001	AD	87	M	10	Not available	U of Miami, Brain Bank	IV	No LBs
C5	Hct ZZT_16_0001	Control	84	M	27.5	Not available	U of Miami, Brain Bank	Neurofibrillary degeneration not present	No LBs
C6	Hct ZZK_16_0002	Control	54	M	26.5	Not available	U of Miami, Brain Bank	Neurofibrillary degeneration not present	No LBs
C7	Hct ZT_16_0004	Control	76	M	30.25	Not available	U of Miami, Brain Bank	Neurofibrillary degeneration not present	No LBs
C8	Hct ZZJ_16_0005	Control	70	M	27.2	8.8	U of Miami, Brain Bank	Neurofibrillary degeneration not present	No LBs
C9	Hct ZR_16_0006	Control	76	M	27.75	Not available	U of Miami, Brain Bank	II	No LBs
C10	Hct YL_16_0007	Control	55	M	26.2	9.2	U of Miami, Brain Bank	Neurofibrillary degeneration not present	No LBs
C11	Hct YP_16_0011	Control	75	M	14.16	Not available	U of Miami, Brain Bank	I	No LBs
C12	HSB4361	Control	77	F	14.7	5.2	Sepulveda	Neurofibrillary degeneration not present	No LBs
C13	HSB5410	Control	81	F	12.5	7.1	Sepulveda	Neurofibrillary degeneration not present	No LBs

C14	HSB4814	Control	70	F	16.1	6.6	Sepulveda	Neurofibrillary degeneration not present	No LBs
C15	HSB4322	Control	88	M	12.4	6.5	Sepulveda	Neurofibrillary degeneration not present	No LBs
C16	HSB4660	Control	73	F	18.5	5.6	Sepulveda	Neurofibrillary degeneration not present	No LBs
P1	PD060	PD	84	M	9	6.8	PD UK Brain Bank	II	5
P2	PD079	PD	78	F	22	7.7	PD UK Brain Bank	I	6
P3	PD102	PD	81	M	16	6.1	PD UK Brain Bank	II	5
P4	PD104	PD	75	M	15	7.1	PD UK Brain Bank	I	5
P5	PD106	PD	75	M	3	8.1	PD UK Brain Bank	II	6
P6	HSB 4353	PD	73	M	15	7.5	Sepulveda	Not available	4
P7	HSB 4355	PD	79	M	12.3	7.9	Sepulveda	III	6
P8	HSB 4530	PD	82	M	13	5.1	Sepulveda	Neurofibrillary degeneration not present	4
P9	HSB4554	PD	83	M	6.7	6.7	Sepulveda	Neurofibrillary degeneration not present	5
P10	HSB 4620	PD	78	M	13	8.4	Sepulveda	III	5
P11	HSB 4653	PD	87	M	11.2	6.3	Sepulveda	Not available	5
P12	HSB 4772	PD	81	M	7.9	6.5	Sepulveda	Neurofibrillary degeneration not present	5
P13	HSB 4775	PD	75	M	13.8	7.2	Sepulveda	III	6

P14	HSB 4919	PD	83	M	16.3	5.4	Sepulveda	IV	6
P15	HSB 5181	PD	88	M	Not available	Not available	Sepulveda	V	6
P16	HSB4884	PD	79	M	21.8	5.7	Sepulveda	Neurofibrillary degeneration not present	5
P17	HSB4560	PD	83	F	13.0	5.2	Sepulveda	Not available	4
P18	HSB5662	PD	78	M	11.6	1.1	Sepulveda	II	5
P19	HSB4205	PD	74	M	11.4	7.7	Sepulveda	II	5
P20	HSB5626	PD	74	M	26.0	3.0	Sepulveda	I	5
PD1	S13840	PD	83	F	18.3	Not available	Harvard Brain Tissue Resource	III	4
PD2	S07203	PD	80	M	13.42	Not available	Harvard Brain Tissue Resource	II	4
PD3	S11972	PD	81	F	10.15	Not available	Harvard Brain Tissue Resource	I	4
PD4	S14356	PD	75	F	7.08	Not available	Harvard Brain Tissue Resource	II	4
PD5	S05264	PD	75	M	19.6	Not available	Harvard Brain Tissue Resource	I	5
PD6	S10570	PD	88	M	15.92	Not available	Harvard Brain Tissue Resource	III	4
PD7	S03822	PD	83	F	24	Not available	Harvard Brain Tissue Resource	I	4
PD8	S06984	PD	71	M	15.67	Not available	Harvard Brain Tissue Resource	I	4

CERAD	Experiments
Not available	Rnase H2 PCR
Not available	Rnase H2 PCR
Not available	Rnase H2 PCR, 8-oxodG, OGG1 activity, Oxo-DIP
Definitie AD	Rnase H2 PCR, 8-oxodG, OGG1 activity, Oxo-DIP
Criteria not met	Rnase H2 PCR, 8-oxodG, OGG1 activity, Oxo-DIP
Criteria not met	Rnase H2 PCR, 8-oxodG, OGG1 activity, Oxo-DIP
Criteria not met	Rnase H2 PCR, 8-oxodG, OGG1 activity, Oxo-DIP
Criteria not met	Rnase H2 PCR, 8-oxodG, OGG1 activity
Criteria not met	Rnase H2 PCR, 8-oxodG, OGG1 activity, Oxo-DIP
Not available	Rnase H2 PCR, 8-oxodG, OGG1 activity, Oxo-DIP
Criteria not met	Rnase H2 PCR, 8-oxodG, OGG1 activity
Not available	Rnase H2 PCR
Not available	Rnase H2 PCR

Not available	Rnase H2 PCR
Not available	Rnase H2 PCR
Not available	Rnase H2 PCR
Not available	Rnase H2 PCR
Not available	Rnase H2 PCR
Not available	Rnase H2 PCR
Not available	Rnase H2 PCR
Not available	Rnase H2 PCR
Not available	Rnase H2 PCR, 8-oxodG, OGG1 activity, Oxo-DIP
Probable AD	Rnase H2 PCR, 8-oxodG, OGG1 activity, Oxo-DIP
Not available	Rnase H2 PCR, 8-oxodG, OGG1 activity, Oxo-DIP
Not available	Rnase H2 PCR, 8-oxodG, OGG1 activity, Oxo-DIP
Probable AD	Rnase H2 PCR, 8-oxodG, OGG1 activity, Oxo-DIP
Not available	Rnase H2 PCR, 8-oxodG, OGG1 activity, Oxo-DIP
Not available	Rnase H2 PCR, 8-oxodG, OGG1 activity, Oxo-DIP
Definite AD	Rnase H2 PCR, 8-oxodG, OGG1 activity, Oxo-DIP

Definite AD	Rnase H2 PCR, 8-oxodG, OGG1 activity
Not available	Rnase H2 PCR
Not available	Rnase H2 PCR
Not available	Rnase H2 PCR
Not available	Rnase H2 PCR
Probable AD	Rnase H2 PCR
Not available	Rnase H2 PCR
Not available	IHC for SYN1 and S42Y antibodies
Not available	IHC for SYN1 and S42Y antibodies
Not available	IHC for SYN1 and S42Y antibodies
Not available	IHC for SYN1 and S42Y antibodies
Not available	IHC for SYN1 and S42Y antibodies
Not available	IHC for SYN1 and S42Y antibodies
Not available	IHC for SYN1 and S42Y antibodies
Not available	IHC for SYN1 and S42Y antibodies
