# Supplementary material for: Transcriptional mutagenesis of α-synuclein caused by DNA oxidation in Parkinson’s disease pathogenesis
Source: Acta Neuropathol. 2023 Sep 23;146(5):685–705. doi: 10.1007/s00401-023-02632-7 (PMC10564827; doi:10.1007/s00401-023-02632-7)
Supplement: Supplementary file 3 — Supplementary file3 (PDF 62 KB) [file 401_2023_2632_MOESM3_ESM.pdf]

	Forward RH Primers Gen 2	Tm (°C)
L38I Wt	5'-GAAAGACAAAAGAGGGTGTT**rC**T/iSpC3/iSpC3/CG-3'	55.8
L38I	5'-GAAAGACAAAAGAGGGTGTT**rA**T/iSpC3/iSpC3/CG-3'	53.8
S42Y Wt	5′-AGGGTGTTCTCTATGTAGGCT**rC**C/iSpC3/iSpC3/AT-3'	59.2
S42Y	5′-AGGGTGTTCTCTATGTAGGCT**rA**C/iSpC3/iSpC3/AT-3'	57.6
H50N Wt	5'-AAACCAAGGAGGGAGTGGTG**rC**A/iSpC3/iSpC3/TT-3'	61.1
H50N	5'-AAACCAAGGAGGGAGTGGTG**rA**A/iSpC3/iSpC3/TT-3'	58.9
A53E Wt	5′-GGAGTGGTGCATGGTGTGG**rC**A/iSpC3/iSpC3/AT-3'	62.5
A53E	5′-GGAGTGGTGCATGGTGTGG**rA**A/iSpC3/iSpC3/AT-3'	60.3
T72K Wt	5'-TGTTGGAGGAGCAGTGGTGA**rC**G/iSpC3/iSpC3/GT-3'	64.1
T72K	5'-TGTTGGAGGAGCAGTGGTGA**rA**G/iSpC3/iSpC3/GT-3'	61.7
T75K Wt	5'-AGCAGTGGTGACGGGTGTGA**rC**A/iSpC3/iSpC3/GT-3'	64.8
T75K	5'-AGCAGTGGTGACGGGTGTGA**rA**A/iSpC3/iSpC3/GT-3'	62.8
S129Y Wt	5'-CAATGAGGCTTATGAAATGCCTT**rC**T/iSpC3/iSpC3/GC-3'	58.4
S129Y	5'-CAATGAGGCTTATGAAATGCCTT**rA**T/iSpC3/iSpC3/GC-3'	56.7
	Reverse Primers	
RP#1	5'-TCTTGCCCAACTGGTCCTTT-3'	59.2
RP#2	5'-TCCACAGGCATATCTTCCAGAAT-3'	58.3

Amplicon size (bp)
216 with RP#1<br>265 with RP#2
204 with RP#1<br>253 with RP#2
180 with RP#1<br>229 with RP#2
169 with RP#1<br>218 with RP#2
113 with RP#1<br>162 with RP#2
104 with RP#1<br>153 with RP#2
92 with RP#3<br>219 with RP#4
with RP#1 plus 49
