# Supplementary material for: Transcriptional mutagenesis of α-synuclein caused by DNA oxidation in Parkinson’s disease pathogenesis
Source: Acta Neuropathol. 2023 Sep 23;146(5):685–705. doi: 10.1007/s00401-023-02632-7 (PMC10564827; doi:10.1007/s00401-023-02632-7)
Supplement: Supplementary file 4 — Supplementary file4 (PDF 90 KB) [file 401_2023_2632_MOESM4_ESM.pdf]

TM position	TANGO aggregation score
A11D	895.362
A17D	838.552
A18D	838.552
A19D	839.535
T22N	895.275
Q24K	899.266
A27E	896.077
A29E	896.077
A30E	896.077
T33K	899.787
L38I	975.842
S42Y	1053.170
T44N	894.376
H50N	943.487
A53E	844.942
T54K	848.246
A56D	843.752
T59N	895.661
Q62K	907.763
T64K	873.526
A69E	905.445
T72K	412.038
T75K	440.083
A76E	447.175
A78D	653.024
Q79K	730.061
T81K	896.937
A85E	901.350
A89E	896.259
A90E	893.840
Q99K	899.724
A107D	896.077
P108T	896.077
Q109K	899.731
L113M	896.077
P117H	896.955
P120H	896.955
A124D	896.077
P128T	896.077
S129Y	896.077
Q134K	899.731
P138T	896.070
A140D	896.070

Familial Mutations	TANGO aggregation score
WT	896.077
WT 1-120	925.632
A18T	881.034
A29S	895.558
A30P	896.077
E46K	901.432
H50Q	949.801
G51D	879.360
A53T	882.468
A53E	844.942
